# A major secretory defect of tumour-infiltrating T lymphocytes due to galectin impairing LFA-1-mediated synapse completion

**DOI:** 10.1038/ncomms12242

**Published:** 2016-07-22

**Authors:** Anne-Elisabeth Petit, Nathalie Demotte, Benoît Scheid, Claude Wildmann, René Bigirimana, Monica Gordon-Alonso, Javier Carrasco, Salvatore Valitutti, Danièle Godelaine, Pierre van der Bruggen

**Affiliations:** 1 Ludwig Institute for Cancer Research, WELBIO and de Duve Institute, Université catholique de Louvain, B-1200 Brussels, Belgium; 2 TIPs, Université Libre de Bruxelles, B-1050 Brussels, Belgium; 3 Department of Oncology, Grand Hôpital de Charleroi, B-6000 Charleroi, Belgium; 4 INSERM, UMR 1043, Centre de Physiopathologie de Toulouse Purpan, F-31024 Toulouse, France

## Abstract

Surface galectin has been shown to contribute to dysfunctions of human tumour-infiltrating lymphocytes (TILs). We show here that galectin-covered CD8 TILs produce normal amounts of intracellular cytokines, but fail to secrete them because of defective actin rearrangements at the synapse. The non-secreting TILs also display reduced adhesion to their targets, together with defective LFA-1 recruitment and activation at the synapse. These defects are relieved by releasing surface galectin. As mild LFA-1 blockade on normal blood T cells emulate the defects of galectin-covered TILs, we conclude that galectin prevents the formation of a functional secretory synapse by preventing optimal LFA-1 triggering. Our results highlight a major secretory defect of TILs that is not revealed by widely used intracellular cytokine immunomonitoring assays. They also provide additional insights into the T-cell response, by showing that different thresholds of LFA-1 triggering are required to promote the intracellular production of cytokines and their secretion.

## Abstract (web summary)

Galectin-3 is a sugar-binding protein that can inhibit antitumour cytotoxic immunity. Here the authors show that Galectin-3 expressed by tumour cells inhibits LFA-1 on cytotoxic lymphocytes, impairing immunological synapse formation, IFNg secretion, and target cell killing.

## Graphical abstract

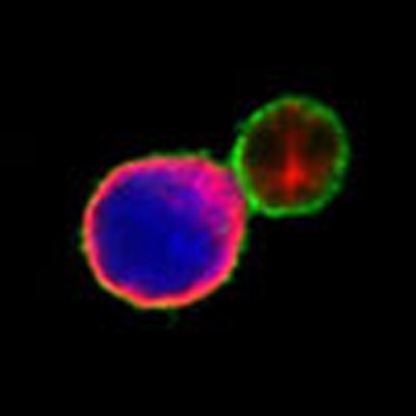

Cancer patients mount T cell responses against specific tumour antigens and specific T cells can infiltrate tumour sites^1^. However, freshly isolated human and murine tumour-infiltrating T lymphocytes (TILs) were observed to be often functionally impaired. After a few hours of *in vitro* culture they partially recovered their function^2^,^3^,^4^. A better understanding of TIL dysfunction is clearly required for improving cancer immunotherapies.

In functional CD8 T cells, recognition of peptide-major histocompatibility complex (MHC) complexes by the T-cell receptor (TCR) triggers a cascade of intracellular signalling events that leads to cytokine production as well as secretion of cytokines and lytic enzymes, which deliver lethal hits to target cells. Activation of T cells implies the formation of a specifically structured communication area at the contact zone known as immunological synapse^5^. Surface receptors and signalling molecules are recruited at the immunological synapse and segregate into concentric rings, the supramolecular activating complexes (SMAC). It was initially believed that the formation of a mature immunological synapse with a characteristic core of TCR surrounded by adhesion molecules was necessary for initiating TCR signalling^5^. Later studies showed that both TCR signalling and calcium fluxes occur before the formation of a mature immunological synapse^6^,^7^, therefore suggesting that stably formed SMAC are rather required to sustain signalling and potentiate full effector T-cell functions^8^,^9^. In addition, the CD8 T cells that secrete cytokines and lytic enzymes have in their immunological synapse a secretory domain, where the release of lytic granules and some cytokines takes place^10^,^11^. Such immunological synapses have been named secretory synapses^12^.

Galectin-1 and galectin-3 could contribute to tumour immunosuppression. They are lectins, that is, sugar-binding proteins, which are mainly secreted by tumour cells and macrophages, but can also be secreted by activated B and TILs (refs 13, 14). Extracellular galectins were reported to bind to various glycosylated T-cell surface receptors^15^,^16^,^17^. By cross-linking glycoproteins at the T-cell surface, they can induce apoptosis of activated T cells^18^,^19^,^20^.

In addition, we have previously shown that the presence of surface galectin contributed to TIL dysfunctions. These CD8 TILs had a low cytotoxicity and a weak ability to secrete cytokines^21^,^22^,^23^, and these defects correlated with the amount of galectin-3 at their surfaces^21^. Treating TILs with an anti-galectin-3 antibody or a galectin competitive binder, for example, *N*-acetyllactosamine (LacNAc), resulted in the detachment of surface galectin-3. On stimulation with beads coated with anti-CD3 and anti-CD28 antibodies, treated TILs had increased cytotoxicity and ability to secrete cytokines such as interferon (IFN)-γ (refs 22, 23). More than 50 fresh CD8 TIL samples were isolated from tumour ascites obtained from patients with various cancers, in particular ovarian and pancreatic carcinomas, and from solid tumours, mostly melanomas. A short LacNAc treatment boosted IFN-γ secretion in 80% of the samples^21^,^23^. More recently, murine TILs were also found to be covered by galectin-3 and improved CD8 T-cell functions were reported in galectin-3-deficient mice^24^.

Through its ability to oligomerize, galectin form glycoprotein-galectin lattices and presumably decreases the mobility of T-cell surface receptors^25^. In TILs covered by galectin-3, we observed a physical dissociation between TCR and CD8 that could be relieved by detaching surface galectin-3 (refs 22, 23). We thus hypothesized that the dysfunction of TILs covered with galectin could be due to low TCR mobility, resulting in defective TCR engagement. This hypothesis was in agreement with previous reports concerning murine T cells, proposing the TCR and CD45 as targets for galectin^15^,^16^. The authors proposed that glycoprotein-galectin lattices would prevent TCR clustering at the immunological synapse and retain phosphatase CD45 in membrane microdomains, where it would interfere with TCR signalling.

We report here that TILs, even when covered with galectin, are able to engage their TCR and produce normal amounts of intracellular cytokines. However, these cytokines are not secreted. We therefore examined in detail the formation of the immunological synapse in freshly isolated human CD8 TILs before and after the removal of galectin with LacNAc and an anti-galectin-3 antibody, as LacNAc is not a galectin-3-specific reagent.

## Results

### Detaching galectin restores TIL function

We previously analysed human TIL functional responses following stimulation with beads coated with anti-CD3 and anti-CD28 antibodies^21^,^23^. To be able to examine the formation of an immunological synapse, we resorted to stimulation with human target cells pulsed with a combination of bacterial superantigens (sAg).

CD8 TILs were isolated from tumour ascites and, without prior *in vitro* expansion, they were treated overnight with either LacNAc, a competitive binder to galectin-3, or an anti-galectin-3 antibody. Both reagents detach galectin-3 from TIL surface^23^, without altering the surface expression of TCR/CD3 complexes, CD8 and LFA-1 (Supplementary Fig. 1b). TILs were then stimulated with Epstein–Barr virus-transformed B (EBV-B) cells pulsed with sAg. IFN-γ secretion was assessed after overnight coculture.

**Figure 1:**
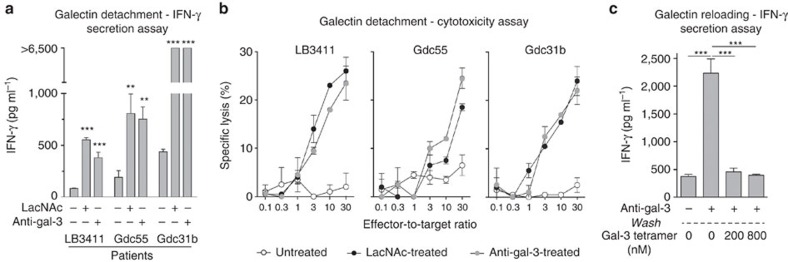
Treating TILs with LacNAc or an anti-galectin-3 antibody boosts their effector functions. CD8 TILs were isolated from ascites (Supplementary Table 1) and treated with LacNAc or an anti-galectin-3 antibody. (**a**) 10,000 T cells were cultured for 20 h with sAg-pulsed EBV-B cells. IFN-γ secretion was measured by enzyme-linked immunosorbent assay (ELISA). Columns represent means±s.d. of triplicates. Unpaired *t*-test, ***P<0.0001. TILs were treated overnight for convenience purposes, but the results obtained after a short TIL treatment of 2 h were similar (data not shown). (**b**) Target cells were pulsed with sAg, ^51^Cr-labelled and incubated with TILs at the indicated ratios. Chromium release was measured after 4 h of coculture. Data represent the mean±s.d. of duplicates. (**c**) CD8 TILs from Gdc55 sample were treated for 2 h with an anti-galectin-3 mAb (10 μg ml^−1^), subsequently removed using anti-rat IgG-coated magnetic beads and a magnet. TILs were reloaded for 30 min with galectin-3 tetramers, and cultured for 20 h with sAg-pulsed EBV-B cells. IFN-γ secretion was measured by ELISA. Columns represent means±s.d. of triplicates.; unpaired *t*-test, ***P<0.001.

Treatment with LacNAc or the anti-galectin-3 antibody significantly increased IFN-γ secretion (Fig. 1a). In total, 40 CD8 TIL samples obtained from patients bearing tumours of different histological origins were treated with LacNAc. IFN-γ secretion was increased more than 2.5-fold in 35 (87%) of the TIL samples. The median value was 5.7-fold (Supplementary Table 1). We conclude that the secretion defect of TILs related to the presence of surface galectin is a very general phenomenon. Remarkably, low amounts of IFN-γ are nevertheless released by untreated TILs, indicating that the secretion is not completely blocked (Fig. 1a). Reloading galectin-3 on TILs, which were previously treated with an anti-galectin-3 antibody, resulted in a decreased IFN-γ secretion on restimulation (Fig. 1c). We did not observe an increase in IFN-γ secretion after LacNAc treatment of blood CD8 T cells obtained from four melanoma-bearing patients and four non-cancerous donors (Supplementary Fig. 2). Noteworthy, culturing blood CD8 T cells in the presence of galectin-3 for 3 days decreased their efficiency for secreting IFN-γ on a further stimulation (Supplementary Fig. 3).

Treatment with LacNAc or the anti-galectin-3 antibody caused significant increase in lysis of sAg-pulsed target cells (Fig. 1b). Enhanced cytotoxicity was paralleled by an increase in the percentage of degranulating TILs, as measured by surface expression of CD107a and b (Supplementary Fig. 4). We previously published that the percentage of cells expressing perforin or granzyme B was equivalent in untreated and LacNAc-treated TILs (ref. 23). As the increase in TIL cytolytic activity cannot be attributed to an increased production of lytic enzymes by LacNAc-treated TILs, it is most probably due to an increased exocytosis of granules.

These results agree with our previous results obtained after TIL stimulation with beads coated with anti-CD3 and anti-CD28 antibodies^21^,^23^.

### Galectin does not disturb the initiation of TCR signalling

Initial TCR engagement by peptide-MHC complexes induces the continuous recruitment of surface TCR towards the contact zone with the target, followed by the internalization of TCR complexes^26^. Staining the TCR-CD3 complexes that are exposed on the surface before and after stimulation provides an estimate of ligand-engaged complexes that are internalized.

**Figure 2:**
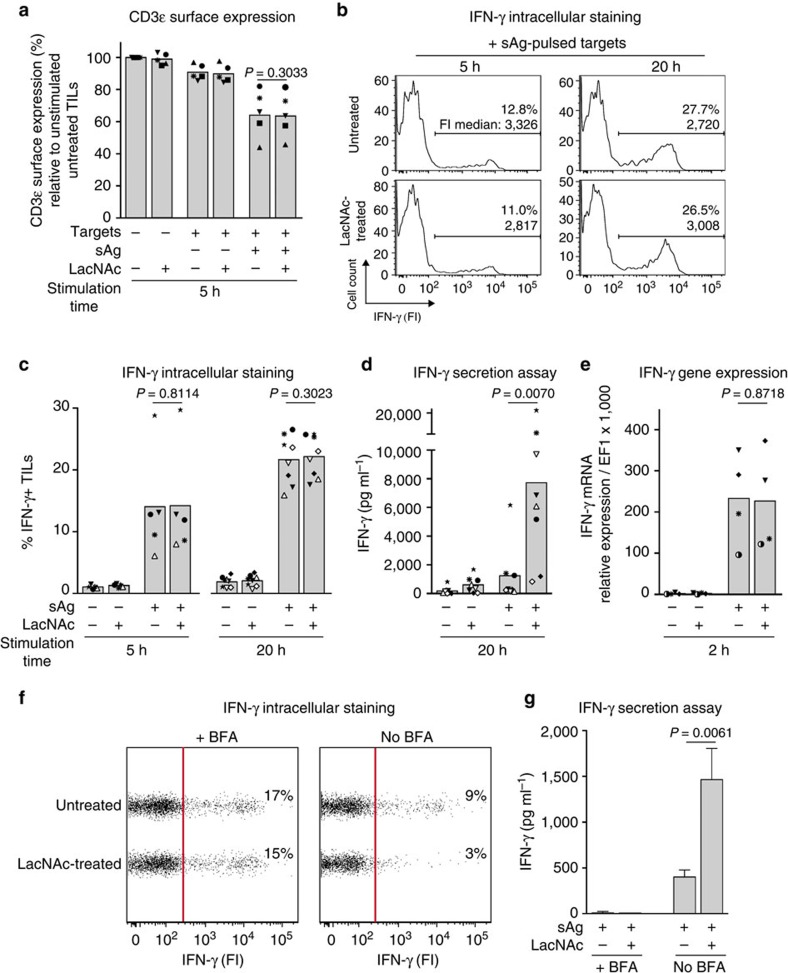
LacNAc treatment is dispensable for TCR internalization and IFN-γ production by TILs. CD8 TILs were isolated from ascites and treated with LacNAc. TILs were cultured with sAg-pulsed EBV-B cells. Each symbol represents TILs from one patient (Supplementary Table 1). (**a**–**e**) Data were obtained in the same experiment for each patient. Columns represent means for several patients. Paired *t*-test. (**a**) Cells were stained with anti-CD3ɛ mAb. Data represent the percentage of CD3ɛ surface expression on TILs, relative to unstimulated conditions. (**b**,**c**) Intracellular staining of IFN-γ in TILs cocultured with sAg-pulsed targets in the presence of brefeldin A. (**b**), Representative histograms obtained from patient Gdc44 (●), % of IFN-γ^+^ TILs and the fluorescence intensity (FI) median of the positive subset are indicated (**c**), % of IFN-γ^+^ TILs obtained from each patient. (**d**) IFN-γ secretion was measured by Bioplex. (**e**) IFN-γ messenger RNA expression was measured by quantitative PCR with reverse transcription. (**f**) After 5 h of coculture in the presence or in the absence of brefeldin A (BFA), TILs from sample Gdc42 were stained for intracellular IFN-γ and analysed by flow cytometry. Data at the right of the red line are considered as positive for IFN-γ staining. (**g**) In the same experiment, IFN-γ release was measured in the supernatants by enzyme-linked immunosorbent assay (ELISA). Columns represent mean±s.d. of triplicates. Unpaired *t*-test. (**f**,**g**) Results are from one representative experiment out of three.

After 5 h of stimulation, TCR-CD3 internalization was equivalent in untreated and LacNAc-treated TILs (Fig. 2a). Untreated TILs were also able to upregulate the CD69 surface activation marker as efficiently as their LacNAc-treated counterparts (Supplementary Fig. 5).

### Galectin does not impact on intracellular cytokine production

Cytokine production following TCR activation was estimated by cytokine intracellular staining of TILs stimulated with sAg in the presence of brefeldin A, which blocks protein secretion by inhibiting vesicular transport from the endoplasmic reticulum to the Golgi apparatus. Cells were analysed by flow cytometry, taking into account both the percentage of cytokine-positive TILs and the median fluorescence intensity of the positive subset (Fig. 2b). Analysis of several TIL samples revealed that untreated TILs contain IFN-γ at levels similar to LacNAc-treated TILs (Fig. 2b,c) despite their markedly lower ability to secrete it (Fig. 2d). Also at very early time points after stimulation (1–2 h), *IFNG* gene expression and IFN-γ intracellular production were similar in untreated and LacNAc-treated TILs. However, LacNAc treatment strongly impacted on IFN-γ secretion, which was detectable from 5 h on (Fig. 2e and Supplementary Fig. 6a–c). LacNAc treatment also boosted interleukin (IL)-2 and tumour-necrosis factor (TNF)α secretion without impacting on their intracellular production (Supplementary Fig. 6d–g). Similar results were obtained with TILs treated with an anti-galectin-3 antibody (Supplementary Fig. 7).

All these results indicate that galectin does not impair intracellular production of cytokines but blocks their secretion. In line with these results, untreated TILs stimulated in the absence of brefeldin A had more intracellular IFN-γ than LacNAc-treated TILs and secreted less IFN-γ (Fig. 2f,g).

### Galectin impairs the completion of secretory synapses

The formation of a secretory synapse involves cytoskeletal rearrangements^11^,^12^. The microtubule-organizing centre (MTOC) polarizes towards the target cell and docks to the plasma membrane at the secretory domain, which is located in the central SMAC, the centre of the immunological synapse^11^,^12^. This MTOC polarization is important to direct secretory granules towards the target^11^,^27^,^28^. Concomitantly, after an early step of polymerization at the synapse, the actin cytoskeleton undergoes rearrangements that result in a gradient where actin is more abundant at the synapse periphery. This later step is usually named ‘actin clearing'^11^,^29^. These actin rearrangements appear necessary for the secretion process, presumably allowing the fusion of secretory granules with the plasma membrane^29^,^30^,^31^.

**Figure 3:**
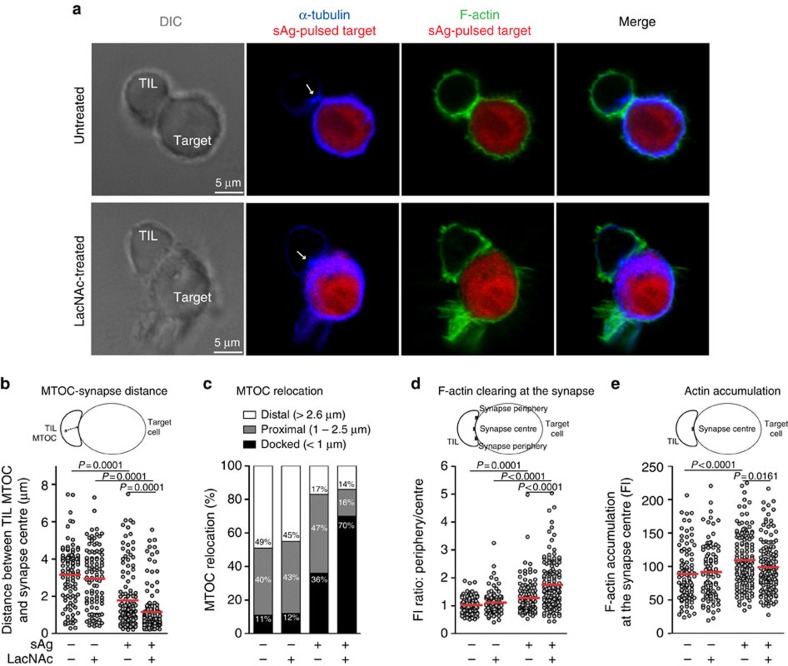
LacNAc treatment of TILs favours secretory synapse completion. CD8 TILs were obtained from sample Gdc31a (Supplementary Table 1). TILs were treated with LacNAc and conjugated with sAg-pulsed EBV-B cells, previously loaded with CMTMR (*red*). (**a**) After 25 min, cells were stained for α-tubulin (*blue*) and F-actin (*green*). TIL MTOCs are indicated by white arrows; original magnification × 63, × 3 zoom. Scale bars, 5 μm. (**b**) MTOC polarization was evaluated by measuring the distance between TIL MTOC and the centre of the TIL-target contact zone. (**c**) The MTOC was considered as ‘docked' to the membrane when it was closer than 1 μm of the centre of the contact zone, as ‘proximal' when it was located between 1 and 2.5 μm, and as ‘distal' when at more than 2.6 μm. (**d**) F-actin clearing at the synapse centre was evaluated by comparing fluorescence intensities (FI) at the synapse periphery and the synapse centre. (**e**) F-actin accumulation was evaluated by measuring FI at the synapse centre. (**b**,**d**,**e**) Each dot corresponds to a TIL-target conjugate; *n*>100; unpaired Mann–Whitney *U* test. Results are from one representative experiment out of two. DIC, differential interference contrast.

We used confocal microscopy to compare the formation of secretory synapses in TILs treated or not with LacNAc. To visualize the MTOC, the microtubules were stained with an anti-α-tubulin monoclonal antibody (mAb) (Fig. 3a). MTOC polarization was evaluated by measuring the distance between the MTOC and the centre of the contact zone with the target. Most TILs, whether treated or not, polarized their MTOC towards their target on conjugation with sAg-pulsed cells (Fig. 3b). We considered that MTOC was ‘docked' to the membrane when it was closer than 1 μm from the centre of the contact zone, ‘proximal' when it was located between 1 and 2.5 μm, and ‘distal' when further than 2.5 μm. While 36% of untreated TILs had a docked MTOC, this percentage increased to 70% in LacNAc-treated TILs (Fig. 3c). A large fraction of untreated TILs had their MTOC in a proximal position, suggesting that galectin prevents the MTOC docking at the synapse.

Actin rearrangements were analysed in the same conjugates. Actin clearing was estimated by comparing the intensities of actin staining at the synapse periphery and the synapse centre. It was more pronounced in LacNAc-treated TILs compared with untreated ones (Fig. 3d). Remarkably, untreated TILs retained their ability to accumulate actin at the synapse centre despite their inability to clear it (Fig. 3e). Thus, LacNAc treatment favoured actin clearing at the TIL immunological synapse. Treating TILs with an anti-galectin-3 mAb also favoured MTOC docking and actin clearing (Supplementary Fig. 8a,b).

Remarkably, actin clearing was correlated with MTOC docking at the secretory synapse in individual TIL-target cell conjugates (Supplementary Fig. 8c). This result is in agreement with the proposal of Griffiths and colleagues that these two events are correlated and therefore could be governed by a unique mechanism^11^.

We also noticed that LacNAc treatment improved TIL spreading on their target (Fig. 3a and Supplementary Fig. 9). Because this could reflect an enhanced adhesion of TILs, we decided to investigate the stability of TIL-target interactions.

### Galectin interferes with the TIL adhesion to their target

**Figure 4:**
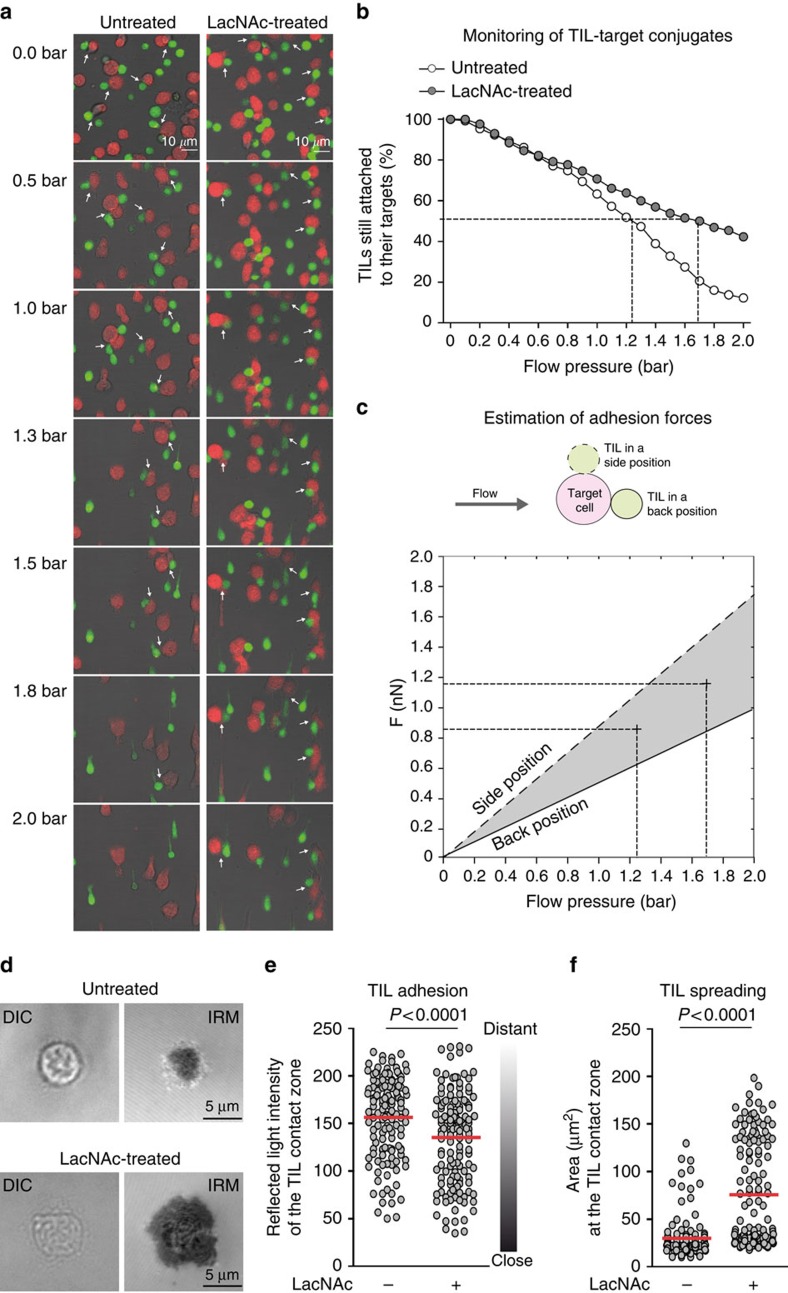
LacNAc treatment increases TIL adhesion to their targets. CD8 TILs from patients LB3450 (**a**–**c**) and Gdc58 (**d**–**f**), Gdc58 (Supplementary Table 1) were treated with LacNAc. (**a**) Monitoring of detachment of TILs (green) from their target (red), under increasing flow pressure. Arrows indicate the conjugates that were selected at 0 bar and used for quantification. See also Supplementary Movies 1 and 2. (**b**) *n*>130 conjugates were examined. (**c**) Forces exerted by the flow on TILs for two different drag coefficients corresponding to their back (solid line) and side (dashed line) positions. The grey area between the two lines represents the range of estimated forces. Forces exerted by the flow on single TILs are shown in Supplementary Fig. 10c,d. (**d**) TILs were allowed to settle on ICAM-1/anti-CD3 mAb-coated slides. After 25 min, cells were observed by IRM. Scale bars, 5 μm. (**e**) Quantification of the intensity of the reflected light and (**f**) of the area of the TIL contact zone. (**d**–**f**) *n*>136; Mann–Whitney *U* test. Results are from one representative experiment out of two. DIC, differential interference contrast; IRM, interference reflection microscopy.

To estimate TIL adhesion forces, red fluorescence-labelled sAg-pulsed target cells were attached to the bottom of a microfluidic chamber. Subsequently, green fluorescence-labelled TILs were allowed to adhere to their targets. A culture medium flow was generated in the chamber and the pressure was progressively increased up to 2 bar. Disruption of TIL-target conjugates (*n*>130) was monitored by fluorescence video microscopy (Fig. 4a,b and Supplementary Movies 1 and 2). At a pressure of 0.6 bar, about 20% of TILs were detached from their targets, whether TILs were treated or not. They were most probably TILs undergoing unspecific interactions. At 2 bar, only ∼12% of untreated TILs still formed conjugates compared with ∼42% of LacNAc-treated TILs. The forces that were experienced by TILs attached to their targets due to fluid stresses were estimated depending on their position on their target (Fig. 4c and Supplementary Fig. 10a,b). The force needed to disrupt a TIL-target conjugate was in the range of the nanonewton (nN). The order of magnitude of its value is compatible with those reported before^32^,^33^. The forces needed to detach 50% of the conjugated TILs were 36% larger for LacNAc-treated cells than for untreated ones (Fig. 4b,c). We concluded that galectin decreases the stability of TIL-target interactions.

The LFA-1 integrin is considered crucial for T-cell adhesion^34^. To examine whether its function could be affected by galectin, CD8 TILs were allowed to settle on slides coated with both recombinant ICAM-1, the main ligand of LFA-1, and an anti-CD3 agonist antibody to induce the recruitment of the integrin. The adhesion of TILs to the slides was evaluated by interference reflection microscopy (IRM). This technique generates black regions in close contact zones and bright regions in distant contact zones. The intensity of the reflected light decreased at the adhesion area of LacNAc-treated TILs, indicating a closer contact with the slide (Fig. 4d,e). The adhesion area, indicative of cell spreading, increased after LacNAc treatment (Fig. 4d,f), again suggesting that TIL-target interactions were disturbed by galectin. Interestingly, when slides were coated with ICAM-1 but no anti-CD3 antibody, TIL adhesion still appeared to be greater after LacNAc treatment (Supplementary Fig. 10e). These results suggested that galectin could disturb LFA-1/ICAM-1 interactions.

### Galectin perturbs LFA-1 triggering at the synapse

**Figure 5:**
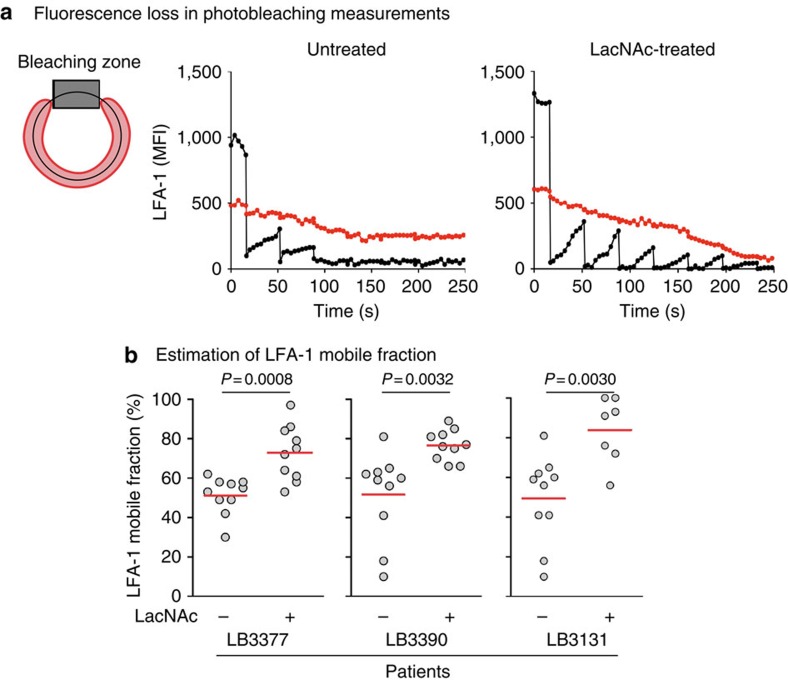
Mobility of LFA-1 at the TIL surface. CD8 TILs were isolated from ascites (Supplementary Table 1) and treated with LacNAc. TILs were stained with an AlexaFluor 488-labelled anti-CD11a mAb. LFA-1 mobile fraction was estimated by using fluorescence loss in photobleaching (FLIP) analysis. (**a**) The extent of fluorescence loss was monitored at distant regions (red) from the beaching zone (black). (**b**) Fluorescence intensities in the measurement region were plotted against time and normalized between 0 and 100%, zero was defined as the smallest value in the data set, 100 as the largest value in the data set. The immobile fraction is determined by the mean of the five lowest intensities. The mobile fraction=1/immobile fraction. Each dot corresponds to a TIL. Unpaired *t*-test. Results were obtained from three independent experiments.

The LFA-1 integrin, which is glycosylated, could be trapped by glycan-galectin lattices at the TIL surface, resulting in a decreased mobility of LFA-1. Its lateral diffusion was estimated at the surface of unstimulated TILs in photobleaching experiments. Treating TILs with LacNAc or an anti-galectin-3 antibody resulted in a significant increase of the mobile fraction of LFA-1, indicating that galectin perturbs LFA-1 lateral diffusion (Fig. 5 and Supplementary Fig. 11).

**Figure 6:**
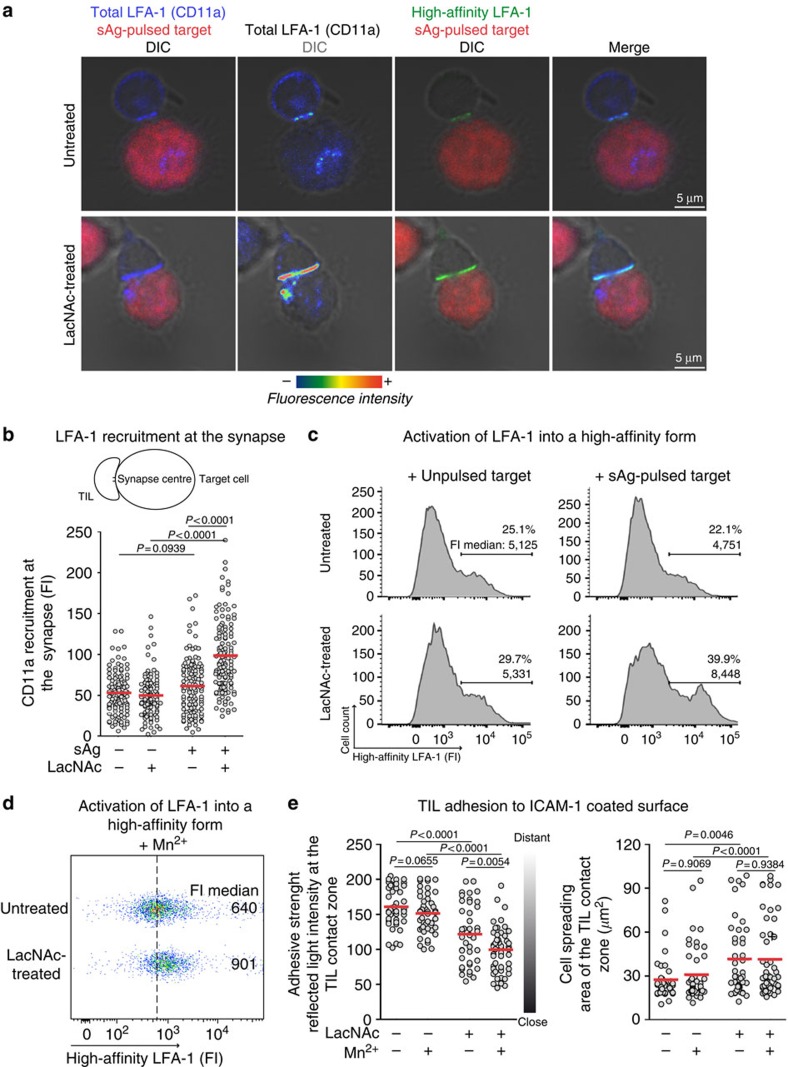
LacNAc treatment improves LFA-1 recruitment at the synapse and its activation into a high-affinity form. CD8 TILs from patients Gdc58 (**a**,**c**–**e**) and Gdc29 (**b**) (Supplementary Table 1) were treated with LacNAc. (**a**) TILs were conjugated with sAg-pulsed EBV-B cells, previously loaded with CMTMR (red). After 25 min, cells were stained for total LFA-1 (CD11a, blue, pseudocolor) and high-affinity LFA-1 (green). Original magnification × 63, × 3 zoom. Scale bars, 5 μm. (**b**) LFA-1 recruitment at the synapse was evaluated by measuring the fluorescence intensity (FI) of the CD11a staining at the centre of the TIL-target contact zone. *n*>110; Mann–Withney *U* test. (**c**,**d**) TILs were loaded with CMFDA and co-cultured with (**c**) EBV-B cells previously pulsed or not with sAg, or (**d**) in presence of Mn^2+^ (0.5 mM). An anti-high-affinity LFA-1 mAb (clone 24) was added simultaneously to the stimuli. After 25 min, cells were washed, fixed and stained with an appropriate secondary antibody coupled to AlexaFluor647. On its own, this antibody does not induce LFA-1 conformational change, though it can stabilize the ICAM-1 bound open conformation. (**c**) The percentage of positive cells and the FI median of the positive subset were estimated for the CMFDA^+^ TILs. (**d**) The FI medians were estimated for the CMFDA^+^ TILs. The dotted line represents the FI median of the untreated TILs. (**e**) TILs were allowed to settle on ICAM-1-coated slides in presence of Mn^2+^ (0.5 mM). After 25 min, cells were observed by IRM. Quantification of the intensity of the reflected light and of the area of the TIL contact zone. *n*>43 per condition; Mann–Whitney U test. Results are representative of two (**a**,**b**–**e**) and three (**c**) independent experiments. DIC, differential interference contrast.

On T-cell stimulation, the LFA-1 integrin is normally recruited to the centre of the synapse, where it accumulates and mediates the adhesion between T cells and targets^5^,^35^. As galectin perturbs LFA-1 lateral diffusion, we hypothesized that it could also interfere with LFA-1 recruitment at the synapse. After 25 min of TIL-target conjugation, we observed by confocal microscopy a higher enrichment of the CD11a subunit of LFA-1 at the immunological synapse of LacNAc-treated TILs compared with untreated TILs (Fig. 6a,b).

LFA-1 ligation to ICAM-1 activates a local signalling pathway, which triggers LFA-1 to adopt a high-affinity extended/open conformation^34^. We thus examined whether galectin could interfere with LFA-1 affinity regulation taking place during TIL-targets interactions by flow cytometry. Using an antibody specific for the high-affinity conformation of LFA-1, we observed that the percentage of positive cells was higher in LacNAc-treated TILs (Fig. 6c). Moreover, the positive subset had a higher fluorescence intensity, probably because of a higher LFA-1 recruitment to the synapse formed with sAg-pulsed targets, where interactions between LFA-1 and ICAM-1 occur (Fig. 6a).

We examined the impact of galectin on the affinity regulation of LFA-1 using manganese, a general integrin activator triggering LFA-1 to adopt a high-affinity conformation. A LacNAc treatment slightly improved this affinity regulation, suggesting that galectin interferes with this local mechanical stimulus (Fig. 6d). Concerning TILs adhesion to ICAM-1-coated surface, manganese treatment slightly increased the adhesive strength, but only for the LacNAc-treated TILs (Fig. 6e). It had no effect on cell spreading. Noteworthy, both adhesive and secretory defects of galectin-covered TILs were rescued by phorbol myristate acetate (PMA) treatment (Supplementary Fig. 12a,b). Considering that PMA does not detach galectin-3 from the cell surface (Supplementary Fig. 12c), we attribute this rescue to the fact that PMA is a very strong stimulus, which, through the cell membrane, diffuses into the cytoplasm, where it activates protein kinase C, omitting the need of surface receptor stimulation.

We concluded that galectin perturbs LFA-1-mediated adhesion by impeding both the recruitment and the affinity regulation at the synapse.

### LFA-1 controls T-cell secretory function

**Figure 7:**
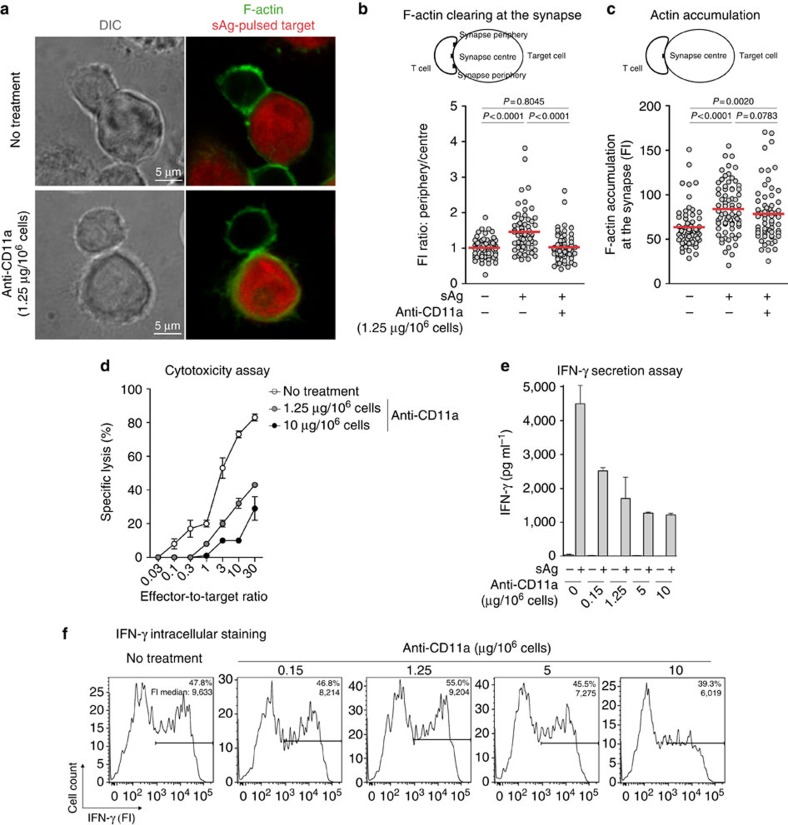
IFN-γ secretion and cytotoxicity depend on LFA-1 function. Blood CD8 T cells from non-cancerous donors LB2050 (**a**–**c**) and LB554 (**d**–**f**), LB554 were treated with an anti-CD11a blocking antibody at the indicated doses. (**a**) T cells were conjugated with sAg-pulsed EBV-B cells, previously loaded with CMTMR (red). After 25 min, cells were stained for F-actin (green). Scale bars, 5 μm. (**b**) Actin clearing at the synapse centre was evaluated by comparing fluorescence intensities (FI) at the synapse periphery and the synapse centre. (**c**) F-actin accumulation was evaluated by measuring FI at the synapse centre. (**b**,**c**) Each dot corresponds to a T-cell-target conjugate; *n*>60; Mann–Whitney *U* test. (**d**) Target cells were ^51^Cr-labelled and incubated with treated T cells at the indicated ratio. Chromium release was measured after 4 h of coculture. Data represent the mean±s.d. of triplicates. (**e**) After 20 h of coculture, IFN-γ secretion was analysed by enzyme-linked immunosorbent assay (ELISA). Data represent the mean±s.d. of triplicates. (**f**) After 20 h of coculture in the presence of brefeldin A, cells were stained for intracellular IFN-γ. Per cent of IFN-γ^+^ T cells and FI medians of the positive subsets are indicated. Results are from one (**a**–**c**), two (**d**) and four (**e**,**f**) independent experiments. DIC, differential interference contrast.

Considering that impaired cytokine secretion by TILs covered by galectin could depend on suboptimal LFA-1-/ICAM-1 interactions, we tried to emulate the phenotype of non-secreting TILs by treating functional blood CD8 T cells with a blocking antibody directed against the CD11a subunit of LFA-1. We used low antibody doses as we intended to block LFA-1/ICAM-1 interactions only partially. Even the lowest dose of this antibody nearly abolished firm adhesion of T cells to ICAM-1-coated slides, indicating inhibition of LFA-1 function (Supplementary Fig. 13a). Antibody-treated cells formed smaller interaction areas with their targets, compared with untreated cells (Fig. 7a and Supplementary Fig. 13b). Blocking LFA-1 ligation to ICAM-1 abolished actin clearing, without affecting the initial step of actin polymerization at the immunological synapse (Fig. 7a–c). The cytotoxic activity of these T cells was also reduced by more than 50%, in agreement with a direct correlation between actin clearing and TIL function (Fig. 7d). Importantly, blocking LFA-1 affected the secretion of IFN-γ without affecting its intracellular production (Fig. 7e,f). Only the highest doses of anti-CD11a antibody slightly decreased the amount of intracellularly produced IFN-γ (Fig. 7f). Similar results were obtained using an LFA-1 small-molecule antagonist, BMS-587101 (Supplementary Fig. 14)^36^. In conclusion, mild LFA-1 blockade seems to emulate the secretory defect observed in galectin-covered TILs.

**Figure 8:**
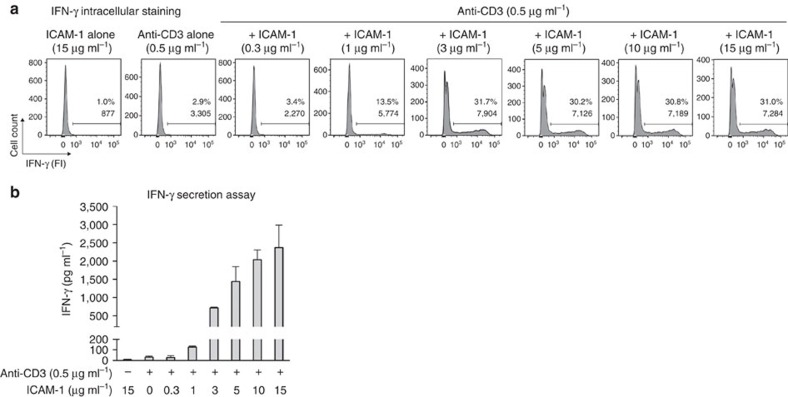
More LFA-1/ICAM-1 interactions are required for IFN-γ secretion than for IFN-γ intracellular production. Blood CD8 T cells from non-cancerous donor LB3442 were plated in wells coated with an anti-CD3 mAb (0.5 μg ml^−1^) and increasing doses of human ICAM-1-Fc. Taking in account this strong stimulus, IFN-γ secretion and production were estimated 3 h after stimulation. (**a**) After stimulation in the presence of brefeldin A, cells were stained for intracellular IFN-γ. (**b**) IFN-γ secretion was measured by enzyme-linked immunosorbent assay (ELISA). Columns are mean±s.d. of duplicates. Results are from one representative experiment out of four.

To further stress the role of LFA-1 in the regulation of cytokine production and secretion, functional blood CD8 T cells were plated in wells coated with an anti-CD3 mAb and increasing doses of recombinant ICAM-1. Although intracellular production of IFN-γ required ICAM-1, it plateaued at 3 μg ml^−1^ (Fig. 8). By contrast, IFN-γ secretion strongly increased from 3 μg ml^−1^ of ICAM-1 on, without reaching a plateau.

This same effect was seen in blood CD8 T-cells stimulated with beads coated with an anti-CD3 mAb and increasing doses of recombinant ICAM-1. The presence of ICAM-1 increased the secretion of IFN-γ without affecting its intracellular production (Supplementary Fig. 15a,b). Actin clearing was more pronounced when T cells were stimulated with beads coated with both anti-CD3 mAb and recombinant ICAM-1, than with anti-CD3 or ICAM-1 alone (Supplementary Fig. 15c).

While both cytokine production and secretion depend on LFA-1/ICAM-1 interactions, many more interactions are required for optimal secretion than for production.

## Discussion

The major finding of our work is that galectin impairs the function of TILs not by abolishing TCR engagement and intracellular production of cytokines, but by impairing cytokine secretion. Non-secreting TILs polarized their MTOC and polymerized actin at the immunological synapse, but their MTOC was not docked and actin was not cleared from the centre of the synapse. Detachment of galectin allowed for these cytoskeleton rearrangements and the completion of the secretory synapse to take place, resulting in an efficient cytokine secretion and cytolytic activity. TILs were stimulated with sAg to examine the formation of the immunological synapse in freshly isolated human CD8 TILs, for which the antigen specificity was unknown. We recently completed measurements of intracellular and secreted IFN-γ in antigen-specific polyclonal T-cells stimulated with peptide-pulsed cells, and obtained similar results: LacNAc treatment did not impact on intracellular production of IFN-γ, but boosted its secretion (Supplementary Fig. 16). Thus, not only TILs but also T-cells stimulated with targets pulsed with an antigenic peptide can exhibit the same dysfunction, that is, an impaired cytokine secretion without defect in intracellular cytokine production.

Several years ago, we published that galectin-3 precludes co-localization of TCRs and coreceptors CD8 at the surface of dysfunctional T cells. Co-localization studies on single cells, which were not in contact with their targets, showed that galectin-glycoprotein lattices diminish the ability of TCR to colocalize to CD8 molecules anchored in membrane rafts of microvilli. We hypothesized that the mere separation of these two receptors is sufficient to explain the functional impairment of these T cells^22^. In contradiction with this hypothesis, we show here that the galectin-glycoprotein lattices are not sufficient to disturb TCR engagement, nor subsequent intracellular cytokine production.

T cells able to produce intracellular cytokines but unable to secrete them have never been described in the context of cancer. However, it is possible to engineer this uncoupling between cytokine production and secretion in murine mast cells and human CD4 T-cells *in vitro*^30^,^37^. Silencing of either of two important actin regulators, namely Coronin1a and Cdc42 RhoGTPase, reduced cytokine secretion without affecting intracellular production. Moreover, a mutation in DOCK8, another regulator of the actin cytoskeleton, is responsible for a fraction of hyper-IgE syndromes^38^,^39^. T cells from patients with this syndrome produce IFN-γ normally but secrete it poorly.

The importance of actin dynamics in the control of secretory granules extrusion is now well established^30^,^31^,^40^,^41^,^42^. Actin was initially thought to be entirely cleared from the synaptic area. It was therefore proposed that actin acts as a barrier preventing granules to reach the plasma membrane. This interpretation has recently been challenged by super-resolution imaging showing the persistence of a residual actin meshwork within the synaptic area^29^,^43^,^44^. The formation of clearances of sufficient size allowed the passage of secretory granules through the actin meshwork. A complete exclusion of actin from the centre of the synapse may thus not be strictly required for secretion. Super-resolution microscopy will be needed to precisely identify the actin rearrangements that are required for T-cell secretion.

We observed a lower stability of TIL/target interactions in the presence of surface galectin. We established that defective adhesion of TILs relied on LFA-1 impairment. On TCR activation, LFA-1 is normally recruited to the synapse where ICAM-1 ligation occurs. To bind ICAM-1, inactive bent LFA-1 molecules must acquire an extended intermediate-affinity conformation. This external conformational change is induced by ‘inside-out' signalling originating in the activated TCR. Subsequent ligation to ICAM-1 triggers a local ‘outside-in' signalling, resulting in the opening of the LFA-1 headpiece and its conversion into a high-affinity conformation^34^. Several hypotheses, which are not mutually exclusive, could explain how galectin affects LFA-1 function. First, galectin appears to trap LFA-1 into glycoprotein-galectin lattices, reducing its lateral diffusion and consequently, its recruitment to the synapse. Galectin could thus reduce the number of LFA-1 molecules converted in their high-affinity conformation via outside-in signalling by preventing their binding to ICAM-1 presented by target cells. Second, galectin could directly lock LFA-1 in a bent or intermediate-affinity conformation, precluding its conversion into a high-affinity conformation. This could explain why LacNAc-treated TILs appeared more sensitive to Mn^2+^ treatment than their untreated counterparts. Third, galectin could affect TCR-mediated inside-out signalling, precluding the extension of LFA-1 in its intermediate-affinity conformation and thus its ligation to ICAM-1. These three mechanisms are not mutually exclusive and could together disturb the integration of signals from the two different platforms that are necessary for efficient integrin functioning.

Our data indicate that the T-cell response is controlled by LFA-1 in a more subtle way than previously thought. Deleting the LFA-1 gene or severly blocking LFA-1/ICAM-1 interactions was previously shown to reduce TCR signalling, as evaluated by Ca^2+^ mobilization and transcription of cytokine genes^45^,^46^,^47^,^48^. Consequently, we observed that cytokines were barely produced intracellularly in blood T-cells stimulated in the absence of any ligands for LFA-1. This confirms that low levels of LFA-1/ICAM-1 interactions are required for initial cell encounter, initiation of TCR signalling and cytokine production. However, this low threshold, which is sufficient to optimize cytokine production, is insufficient to promote the secretion. It is highly probable that a higher threshold has to be reached at the site of the immunological synapse via the recruitment and the activation of the integrin.

Both in galectin-covered TILs and in LFA-1-blocked blood T cells, we observed that secretory synapse formation was arrested at the early stage, that is, with actin polymerized across the synapse. We concluded that LFA-1 controls secretion by driving the late stage of the secretory synapse formation, that is, actin clearing. Similar arrest in synapse formation was observed by Griffiths and colleagues in murine CD8 T-cells deficient for Lck or with an inactive Zap-70 (refs 41, 42, 49). Considering that Lck and Zap-70 participate in LFA-1 signalling induced by ICAM-1 ligation^50^, these results strengthen our conclusion. Further investigation will be needed to determine the mechanisms controlling the early stages of secretory synapse formation.

Our results illustrate the importance of stable adhesion in the control of T-cell cytotoxicity. In agreement, the group of Huse highlighted the importance of the adhesive strength exerted at the synapse in the control of secretion^51^. They showed that cytotoxic T-cells coordinate synaptic force exertion and lytic granule release. We showed that non-secreting TILs were unable to spread and adhere firmly to their target, because of a defective LFA-1 function. Consistently, non-lytic murine CD8 TILs showed defective LFA-mediated adhesion to their targets^52^. Reduced cytotoxicity of a human CD8 T-cell clone was also observed in the presence of an anti-LFA-1 antibody^53^. However, this was not due to impaired lytic granule secretion, as measured by the release of serine esterase. The authors proposed that the main role of LFA-1 in cytotoxicity is to mediate very tight contacts between membranes of target cells and T cells to avoid mistargeting of the released granules. It is conceivable that the formation of tight contacts necessitates even stronger LFA-1/ICAM-1 interactions than those required for secretion. Consequently, the presence of galectin on TILs may also decrease TIL cytotoxicity by disturbing LFA-1-mediated tight contacts between TILs and their targets.

**Figure 9:**
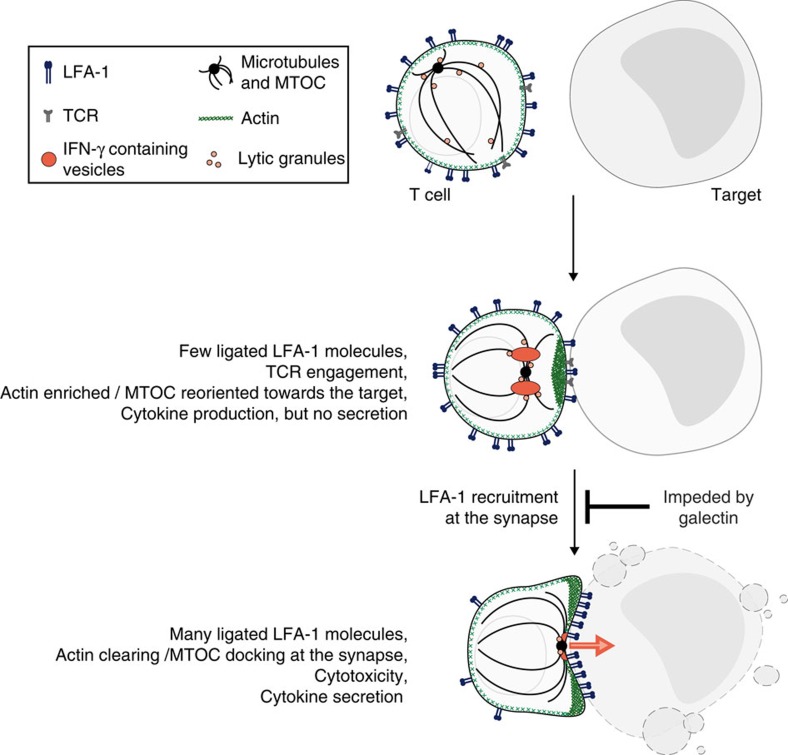
Galectin prevents the formation of a functional secretory synapse by preventing optimal LFA-1 triggering. Activation of a T cell, upon contact with its target, implies the formation of a specifically structured communication area at the contact zone known as immunological synapse. Surface receptors such as the TCR and LFA-1 are recruited at the immunological synapse. The microtubule-organizing centre (MTOC) polarizes towards the target cell and docks to the plasma membrane. Concomitantly, after an early step of polymerization at the synapse, the actin cytoskeleton undergoes rearrangements that result in a gradient where actin is more abundant at the synapse periphery. This later step is usually named 'actin clearing'. These actin rearrangements appear necessary for the secretion process, presumably allowing the fusion of cytokine-containing vesicles and lytic granules with the plasma membrane. Galectin impairs the function of TILs not by abolishing TCR engagement and intracellular production of cytokines, but by impairing cytokine secretion. Galectin-covered TILs polarize their MTOC and polymerized actin at the immunological synapse, but their MTOC was not docked and actin was not cleared from the centre of the synapse. Detachment of galectin allowed for these cytoskeleton rearrangements and the completion of the secretory synapse to take place, resulting in efficient cytokine secretion and cytolytic activity.

We conclude that the entrapment of LFA-1 in glycoprotein-galectin lattices, resulting in the lack of its recruitment at the immunological synapse, provides a sufficient explanation for the lack of secretory ability observed in most TIL samples (Fig. 9). However, this does not exclude that other effects due to galectin could contribute to the lack of secretion. These effects could include the ability to impair the mobility of other surface molecules, such as CD28, which binds to B7 molecules. Several observations suggest indeed that CD28 could play a LFA-1-like role in driving cytoskeleton rearrangements^41^,^54^.

From a clinical point of view, our data stress the danger of evaluating the functional status of T cells exclusively by intracellular cytokine staining, a widely used immunomonitoring assay. T cells accumulating intracellular cytokines on stimulation may nevertheless be dysfunctional, like the TILs described here. As mentioned earlier, the inability to secrete cytokines and lytic enzymes is encountered in TILs present in a large fraction of cancer patient samples^21^,^23^. The positive news for cancer immunotherapy is that the secretory dysfunction of most of these TILs can be relieved by galectin antagonists. Some of them such as GM-CT-01 and GCS-100 are available for human trials^21^,^23^. Clinical strategies involving antibodies preventing inhibitory signals of T-cell activation, such as CTLA-4 and PD-1, or anti-tumoural vaccination could benefit from their combination with galectin antagonists.

## Methods

### T cells and cell lines

The EBV-B cell lines CP50-EBV and LG2-EBV were obtained several years ago in our laboratory by immortalization of blood B cells with EBV, using a published procedure^55^. Cells were cultured in Iscove's modified Dulbecco's medium (IMDM; Life Technologies–Carlsbad, CA, USA) supplemented with 10% foetal calf serum, 0.24 mM L-asparagine, 0.55 mM L-arginine, 1.5 mM L-glutamine (AAG), 100 U ml^−1^ penicillin and 100 μg ml^−1^ streptomycin. Patient-derived tumour ascites and blood cells from haemochromatosis patients were collected after approval by Institutional Review Boards of all collaborating institutions. CD8 TILs and blood CD8 T cells were sorted by a negative selection strategy (Miltenyi Biotec–Bergisch Gladbach, Germany) from the CD2-positive cells selected by rosetting from the ascites using sheep red bloods cells^23^. In some experiments, freshly isolated blood CD8 T cells were first cultured in IMDM, 10% human serum (HS) and 25 U ml^−1^ rhIL-2 (Chiron) in the presence of irradiated LG2-EBV cells, which were previously pulsed with 10 ng ml^−1^ of a cocktail of bacterial sAg: toxic shock syndrome toxin-1, *Staphylococcus aureus enterotoxin A* and *Staphylococcus aureus enterotoxin B* (all from Sigma-Aldrich–Saint-Louis, MO, USA). These T-cell lines were used after 1–2 weeks in culture.

### Treatment of T cells with galectin antagonists

Freshly isolated CD8 TILs and blood CD8 T cells were suspended at 1 × 10^6^ ml^−1^ in IMDM 2% HS and AAG and incubated overnight at 37 °C with LacNAc (5 mM; Elicityl–Crolles, France or Carbosynth–Compton, UK) or an anti-galectin-3 antibody (10 μg ml^−1^; Mabtech–Nacka Strand, Sweden). TILs were diluted 3–10-fold before being used in functional assays. Unless indicated in the figures, TILs were treated overnight. This was only done for convenience purposes. The results obtained after a short TIL treatment of 2 h were comparable to those obtained after overnight treatment, as reported in our previous publications^21^,^23^.

### Galectin-3 tetramer obtention and cell reloading

A cDNA coding for the human galectin-3 was generated by PCR with reverse transcription on total RNA extracted from a melanoma cell line. The cDNA was used as template to amplify by PCR the sequence coding for galectin-3 (amino acids M_1_-I_250_) fused to the C-terminal tag: SG(H)6SC. The PCR product was cloned into a derivative of plasmid pET9 (Novagen, Merck Millipore, Darmstadt, Germany). The soluble protein was expressed in *E. coli* BL21-AI (Invitrogen, Thermo Fisher Scientific, Waltham, MA, USA) and purified by affinity chromatography on α-lactose agarose (Sigma-Aldrich–Saint-Louis, MO, USA). The purified galectin-3 was biotinylated on the C-terminal cysteine with maleimide biotin (EZ-Link maleimide-PEO2-biotin; Thermo Fisher Scientific, Waltham, MA, USA). Galectin-3 tetramers were obtained by mixing the biotinylated galectin and recombinant streptavidine (Roche Applied Science, Mannheim, Germany) with a molar ratio of four to one.

Freshly isolated CD8 TILs were treated for 2 h with anti-galectin-3 mAb, subsequently removed using anti-rat IgG-coated magnetic beads and a magnet. TILs were reloaded with galectin-3 tetramers (200–800 nM) for 30 min at 37°C, washed and tested for their function. Galectin-3 tetramer reloading was controlled by a galectin extracellular staining analysed by flow cytometry.

Freshly isolated blood CD8 T cells were cocultured with anti-CD3/CD28 coated beads at ratio 3:1, in the presence of a galectin-3 tetramer (300–800 nM). After 3 days, T cells were washed and assessed for their function.

### Labelling of surface galectins

Cells obtained from ascites (total, not purified) were treated for 15 min at 4 °C with an FcR blocking reagent (Miltenyi Biotec) and were incubated for 2 h at 37 °C under agitation with LacNAc (5–15 mM) or an anti-galectin-3 antibody (10–30 μg ml^−1^; Mabtech). Cells were washed before being stained with an anti-galectin-3 coupled to biotin (5 μg ml^−1^, clone M3/38; Biolegend–San Diego, CA, USA) followed by phycoerithryn (PE)-coupled NeutrAvidin (1.25 μg ml^−1^; Life Technologies) and with an allophyco-cyanin (APC)-coupled anti-CD8ß (clone 2ST8.5H7, Pharmingen–San Diego, CA, USA). Cells were fixed in 1% paraformaldehyde (PFA) and acquired on a FACSFortessa flow cytometer (Becton Dickinson–Franklin Lakes, NJ, USA). Galectin-3 staining at the surface of CD8^+^ TILs was analysed using software FlowJo (Tree Star–Ashland, OR, USA).

### Cytokine secretion assays

A total of 10,000 T cells were plated in round-bottom microwells with 10,000 CP50-EBV cells previously pulsed with 1 μg ml^−1^ of sAg. Cytokines were measured by enzyme-linked immunosorbent assay in the supernatants of 20 h cocultures using Biosource Cytoset reagents (Life Technologies; IFN-γ) or by multiplex using Bio-Rad BioPlex system (IFN-γ, IL-2 and TNF-α).

### Cytotoxicity assay

CP50-EBV cells were pulsed with sAg and labelled with 50 μCi of Na[^51^Cr]O_4_. T cells were added to the targets, and chromium release was measured after 4 h of incubation.

### Degranulation assay

T cells were plated with sAg-pulsed CP50-EBV cells at ratio 1:1, in presence of fluorescein isothiocyanate-coupled anti-CD107a and anti-CD107b antibodies (clones H4A3 and H4B3, 1:100, BD Pharmigen) and brefeldin A (10 μg ml^−1^; Sigma Aldrich). After 5 h of coculture, cells were washed and stained with APC-coupled anti-CD8ß mAb (clone 2ST8.5H7, BD Pharmingen). Cells were fixed in 1% PFA. Data were acquired on a FACSFortessa and analysed using software FlowJo.

### Immunofluorescence labelling for flow cytometry

T cells were plated with sAg-pulsed CP50-EBV cells, previously loaded for 15 min with 0.5 μM 5-chloromethylfluorescein diacetate CellTracker green CMFDA (Molecular Probes–Eugene, OR, USA) at ratio 1:1. Cells were conjugated by 1 min centrifugation. After 5 h of coculture, TCR internalization was measured by extracellular staining of the CD3ɛ subunit with AlexaFluor 647-coupled anti-CD3ɛ (clone UCHT1, BD Pharmingen). CD69 was stained with a PE-coupled anti-CD69 mAb (clone L78, BD Pharmingen). For cytokine production assays, brefeldin A (5 μg ml^−1^; Sigma-Aldrich) was added after the first hour of coculture. After an additional 3–18 h of incubation, cells were fixed in 1% PFA, permeabilized with 0.1% saponin and stained with PE-coupled anti- IFN-γ (clone B27), APC-coupled anti-IL-2 (clone MQ1-17H12) and AlexaFluor 700-coupled anti-TNFα (clone MAb11; all from BD Pharmingen). Cells were acquired on a FACSFortessa (BD Biosciences–San Jose, CA, USA). CD3ɛ surface expression, IFN-γ or IL-2 or TNFα-positive subset were estimated for the CMFDA-negative population, using software FlowJo. For the detection of the high-affinity form of LFA-1, T cells were stained with 0.5 μM CMFDA and stimulated with sAg-pulsed B cells in presence of antibody 24, which is specific for the high-affinity conformation of LFA-1 (2 μg ml^−1^, clone 24, Abcam–Cambridge, UK). After 25 min of coculture, cells were washed, fixed and stained with an AlexaFluor647-coupled secondary antibody. High-affinity LFA-1 staining was estimated for CMFDA-positive cells. Data were acquired on a FACSFortessa and analysed using software FlowJo.

### Quantitative PCR with reverse transcription for IFN-γ

TILs were conjugated with sAg-pulsed CP50-EBV cells at ratio 1:1 by 1 min centrifugation. Total RNA from 2 h cocultures was prepared with Macherey-Nagel Nucleospin RNA kit and reversed transcripted with Moloney Murine Leukemia Virus Reverse Transcriptase (MMLV-RT) (Life Technologies). PCR amplifications were performed in a final volume of 25 μl with 0.6U of HotGoldStar DNA polymerase (Eurogentec–Seraing, Belgium), 300 nM of each primer, 160 nM of probe, 200 μM of dNTP and 5 mM of MgCl_2_ and run on an ABIPrism 7700 Sequence Detector (Applied Biosystems–Waltham, MA, USA) under standard conditions: 94 °C for 10 min, 40 cycles of 94 °C for 15 s and 60 °C for 1 min. Reactions were performed in technical duplicates for each biological duplicate. Primers can be found in Table 1.

### Immunofluorescence labelling for confocal microscopy

TILs were conjugated with sAg-pulsed CP50-EBV cells previously stained with 5-(and 6)-(((4-chloromethyl)benzoyl)amino)tetramethylrhodamine (CMTMR; 1 μM; Molecular Probes) at ratio 1:1 by 1 min centrifugation. After 25 min of coculture on slides coated with poly-D-lysine (50 μg ml^−1^; Sigma-Aldrich), cells were fixed in 3% PFA, permeabilized with 0.1% saponin in PBS, 3% bovine serum albumin (BSA) and HEPES, and stained with AlexaFluor 488 phalloidin (Molecular Probes) or one of the following antibodies: anti-CD11a (clone EP1285Y, Abcam), anti-α-tubulin (clone DM1A, Sigma-Aldrich). Staining for γ-tubulin (a microtubule nucleator used to detect centrosome position) detects the position of centrosome in cells more precisely than staining for α-tubulin (to detect microtubule organization). Nevertheless it has been shown that in TILs MTOC positioning as indicated by α-tubulin staining co-localizes with centrosome staining^56^. Primary antibodies were followed by appropriate fluorochrome-coupled anti-species isotype specific Ab (Molecular Probes). The samples were mounted in ProLong Gold (Molecular Probes). Random acquisition of T-cell-target conjugates was performed using a Zeiss LSM 510 confocal microscope with a × 63 NA1.4 Plan-Apochromat oil immersion objective (Carl Zeiss–Oberkochen, Germany). Images were processed using software ImageJ (National Institute of Health, USA).

### Interference reflection microscopy

Chambered coverslips (Labtek, Nunc–Roskilde, Denmark) were coated overnight at 4 °C with recombinant human ICAM-1-Fc (R&D Systems–Minneapolis, MN, USA) and anti-CD3 (clone OKT3, Mabtech) diluted at 3 μg ml^−1^ and 1 μg ml^−1^ in PBS, respectively, and blocked with PBS 3% BSA. T cells were allowed to settle for 25 min at 37 °C and non-attached T cells were removed by gentle washing. Cells were analysed by IRM using a Zeiss LSM 510 confocal microscope with a × 63 NA1.4 Plan-Apochromat oil immersion objective. IRM images were obtained using the 633-nm laser line in conjunction with a FT 561 dichroic and an open emission filter. In other experiments, coverslips were coated with only human ICAM-1-Fc and the T cells were treated with 0.5 mM of Manganese (II) chloride tetrahydrate (Mn^2+^, Sigma Aldrich-Diegem, Belgium) in the following buffer: 20 mM Hepes, 140 mM NaCl and 2 mg ml^−1^ of glucose.

### Image quantification

The intensity of CD11a and F-actin stainings were analysed using the Linescan Function of software MetaMorph (Universal Imaging–Bedford Hills, NY, USA). A line of reference of ∼0.5 μm was drawn at the region of interest. This line was drawn inside the T cell to exclude fluorescence originating from the target. The software calculates the average intensity along this line of reference for 12 pixels of width. The regions were defined as shown in Figs 3, 6 and 7. The intensities were measured at the ‘synapse centre' and in ‘peripheral synapse' regions of one T cell, using the same line of reference. To estimate the adhesion status of T cells, each cell was considered as a region, carefully drawn around the IRM images. Each region was analysed for area and brightness (MetaMorph Software). The polarization of the MTOC of the T cell towards its target was evaluated by measuring the distance between MTOC and the centre of the T-cell-target contact zone, using the profile function of the Zeiss LSM Browser software.

### Fluorescence loss of photobleaching experiments

Cells (2 × 10^5^) were washed in Iscove Medium, and resuspended in 250 μl of Iscove medium in poly-D-lysine-coated Labtek chambered coverslips (Nunc). After 30 min of incubation at 37 °C, adherent cells were washed in 250 μl of 0.2% BSA, and covered with 250 μl of AlexaFluor 488-labelled anti-CD11a antibody (HI111; Biolegend) in PBS/BSA. After 30 min of incubation on ice, cells were washed and covered with 500 μl of pre-warmed Iscove Medium. Experiences were performed at 37 °C on a confocal microscope (LSM 510; Carl Zeiss) using a × 63 objective and the 488-nm line. Two regions were defined: a rectangular region defined as the bleaching zone and a polygonal region on the remaining cell surface. We measured the extent of fluorescence loss outside the rectangular bleaching zone as a consequence of photobleaching within the rectangle. Regions were sequentially scanned with the laser at 2% transmission, the time lapse between each image was 4 s. Bleaching was operated after the first five images by illumination with the laser at 100% transmission for 10 iterations; every 20 images, the bleaching zone was again illuminated at 100% transmission. Fluorescence intensities in the measurement region were plotted against time and normalized between 0 and 100%, 0 was defined as the smallest value in the data set, 100 as the largest value in the data set. The immobile fraction is determined by the mean of the 5 lowest intensities. The mobile fraction=1/immobile fraction.

### LFA-1 blockade

T cells were pretreated for 30 min at room temperature in IMDM 10% HS AAG in presence of an anti-human CD11a blocking antibody (clone HI111, BD Pharmingen) or LFA-1 small molecule antagonist BMS-587101 (Key Organics–Camelford, UK).

### T-cell stimulation in anti-CD3/ICAM-1-coated wells

Maxisorp flat-bottom 96-well plates were coated overnight at 4 °C with 50 μl of anti-CD3 mAb (Clone OKT3, Mabtech) diluted at 0.5 μg ml^−1^ in PBS with increasing doses of recombinant human ICAM-1-Fc (0.3–15 μg ml^−1^; R&D Systems). T cells were cultured at 10^5^ cells per well for 3 h to test their ability to produce or secrete cytokines.

### Assessment of TIL adhesion under flow stress conditions

Rhombic chamber chips eP2 in zeonor (microfluidic, ChipShop–Jena, Germany) with the height *h*=200 μm, and the width *w*=2,600 μm were coated with poly-D-lysine (10 μg ml^−1^). sAg-pulsed CP50-EBV cells loaded with CMTMR were let to attach to poly-D-lysine for 20 min. TILs were loaded with carboxyfluorescein succinimidyl ester (Molecular Probes) and allowed to interact for 30 min with the targets. A flow of pre-warmed medium supplemented with 10% HS, AAG and HEPES were generated using the flow controller MFCS-EZ (Fluigent–Villejuif, France) by progressively applying a pressure up to 2 bar, by steps of 0.1 bar each. The flow rate (*Q*) was measured simultaneously using the XL Flow Unit (Fluigent) going up to 5 ml min^−1^, allowing to determine the hydraulic resistance of the circuit through the Hagen–Poiseuille law, *P*=*R*_hyd_*Q*, leading to *R*_hyd_=1.45 ml min^−1^ per bar. The rhombic chamber was placed in a thermalized chamber at 30 °C under a fluorescence microscope (LSM 510, Carl Zeiss) to monitor TIL-target adhesion in real time. Isolated TIL-target conjugates were randomly chosen on the first images of the movies (0 bar). Each conjugate was examined to determine at which pressure the T cell detached from the target.

Numerical simulations of the Navier-Stokes equations for incompressible fluids have been performed using the software COMSOL Multiphysics (COMSOL–Stockholm, Sweden) with the Microfluidic module. Modelling cells by rigid spheres, stationary flow structures around TIL-target conjugates attached to the bottom wall of a channel have been computed and the corresponding drag forces experienced by the cells have been post-processed. The computational domain is a cube of 100 μm edge, as illustrated in Supplementary Fig. 10a,b. The flow is aligned with the x-direction, except in the vicinity of the cells. The boundary conditions are: (i) no-slip at the bottom boundary, representing the channel wall, and on the spheres; (ii) symmetry condition at the top boundary, located at half the height of the channel; (iii) developed laminar flow at the inlet with constant flow rate; (iv) constant pressure at the outlet, which is set to zero without loss of generality; and (v) symmetry conditions at the lateral sides, assuming they are far enough from the cells. The target cell is centred in the computational domain and put in contact with the floor. The TILs are placed in contact with both the target and the floor. Several positions of the TILs around the target have been considered but only results for TILs aside and behind the target are reported (Fig. 4c and Supplementary Fig. 10a,b). These positions correspond to the minimum and the maximum forces exerted by the flow on TILs. For validation purpose, computations with a TIL alone have also been performed. The drag force in this case was always larger than in presence of the target cell, showing the mutual screening effect of cell conjugates. For computations, we have taken average diameters evaluated by microscopy on a large number of TILs and targets, leading to d_*T*_=6.5 μm for the TIL and d_*B*_=9.5 μm for the target. Results are plotted in Supplementary Fig. 1D, having used the viscosity and density of the culture medium at 30 °C, that is, respectively *μ*=8.4 × 10^−4^ Pa.s and *ρ*=10^3^ kg m^−1^ (ref. 57).

The linear behaviour between the force and the applied pressure in all cases is reminiscent of Stokes flow (the maximum Reynolds number obtained for *P*=2 bar is ∼25, which confirms that the flow is always laminar). Linear drag-force/shear-flow relationship has been obtained by O'Neill in the case of a single-spherical cell in contact with a plane^58^. Brooks and Tozeren^59^ extended this study for an array of spherical cells. They found that the cell could be considered to be single if the separation distance between neighbouring cells was larger than five times the cell diameter (our computational domain is ∼10 times the cell diameter). They also found that the Poiseuille flow was macroscopically preserved for a channel height 7.5 times larger than the sphere diameter (our computational and experimental channel height is ∼20 times the cell diameter). Following these authors, we conveniently express the fluid force *F* exerted by the flow on a sphere in the following form:


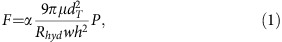


where *α* is the dimensionless drag coefficient, and the other parameters have been previously defined and should be expressed in SI units. For a single sphere (Supplementary Fig. 10d, solid line), we find *α*=1.68, actually very close to O'Neill's analytical result (Supplementary Fig. 10d, dashed line) of *α*=1.70. For a TIL aside the target (Fig. 4c, dashed line), we find *α*=1.31, while for a TIL behind the target (Fig. 4c, solid line) we find *α*=0.74. Note that the drag coefficient is not only a function of the TIL position around the target but also a function of the target diameter. It furthermore depends on the shapes of both the TIL and the target. It is not the purpose here to explore all configurations, but it is worth mentioning that Boulbene *et al*.^60^ have analysed spheroid-shaped cells for varying aspects ratio and orientation, and have shown that the drag force always remains comparable to a sphere, with a difference not larger than a factor 2.

In conclusion, given the variety of configurations, shapes and orientations of TIL-target conjugates, as well as the presence of neighbouring conjugates in the experiments, the computed forces on conjugated TILs presented in Fig. 4c (grey area) are only estimates, yet with orders of magnitude compatible with values reported in the literature^32^,^33^. Although absolute values of adhesion forces can only be obtained qualitatively, quantitative comparison of adhesion forces between treated and untreated cells is still possible, thanks to the linear behaviour of equation (1). This is what we have done in the ‘Results' section.

### Data availability

The data that support the findings of this study are available from the corresponding author on request.

## Additional information

**How to cite this article:** Petit, A.-E. *et al*. A major secretory defect of tumour-infiltrating T lymphocytes due to galectin impairing LFA-1-mediated synapse completion. *Nat. Commun.* 7:12242 doi: 10.1038/ncomms12242 (2016).

## Supplementary Material

Supplementary Information Supplementary Figures 1-16 and Supplementary Table 1

Supplementary Movie 1 Monitoring of untreated TIL conjugates under flow stress conditions. Untreated CD8 TILs from patient LB3450 (Supplementary Table 1) were stained with CFSE (green) and allowed to interact for 30 min with CMTMR (red)-loaded sAg-pulsed B cells. A flow of prewarmed medium was generated by progressively increasing the pressure up to 2 bar, by steps of 0.1 bar. The movie starts at 0.5 bar. TIL detachment from their targets was monitored by video microscopy. Scale bar represents 10 μm. Frames were collected at 4- second intervals. Display rate: 15 frames/second.

Supplementary Movie 2 Monitoring of LacNAc-treated TIL conjugates under flow stress conditions. LacNAc-treated CD8 TILs from patient LB3450 (Supplementary Table 1) were stained with CFSE (green) and allowed to interact for 30 min with CMTMR (red)-loaded sAg-pulsed B cells. A flow of pre-warmed medium was generated by progressively increasing the pressure up to 2 bar, by steps of 0.1 bar. The movie starts at 0.5 bar. TIL detachment from their targets was monitored by video microscopy. Scale bar represents 10 μm. Frames were collected at 4-second intervals. Display rate: 15 frames/second.

## Figures and Tables

**Table 1:** Primers used for quantitative PCR with reverse transcription.

**Gene**	**Primer type**	**Sequence (5′–3′)**
EF1	Sense primer	GCT TCA CTG CTC AGG TGA T
	Antisense primer	GCC GTG TGG CAA TCC AAT
	FAM-TAMRA probe	AAA TAA GCG CCG GCT ATG CCC CTG
IFN-γ	Sense primer	CTA ATT ATT CGG TAA CTG ACT TGA
	Antisense primer	CGA AAC AGC ATC TGA CTC CTT
	FAM-TAMRA probe	TCC AAC GCA AAG CAA TAC ATG AAC TCA TCC
