# Supplementary material for: A major secretory defect of tumour-infiltrating T lymphocytes due to galectin impairing LFA-1-mediated synapse completion
Source: Nat Commun. 2016 Jul 22;7:12242. doi: 10.1038/ncomms12242 (PMC4961845; doi:10.1038/ncomms12242)
Supplement: Supplementary Information — Supplementary Figures 1-16 and Supplementary Table 1 [file ncomms12242-s1.pdf]

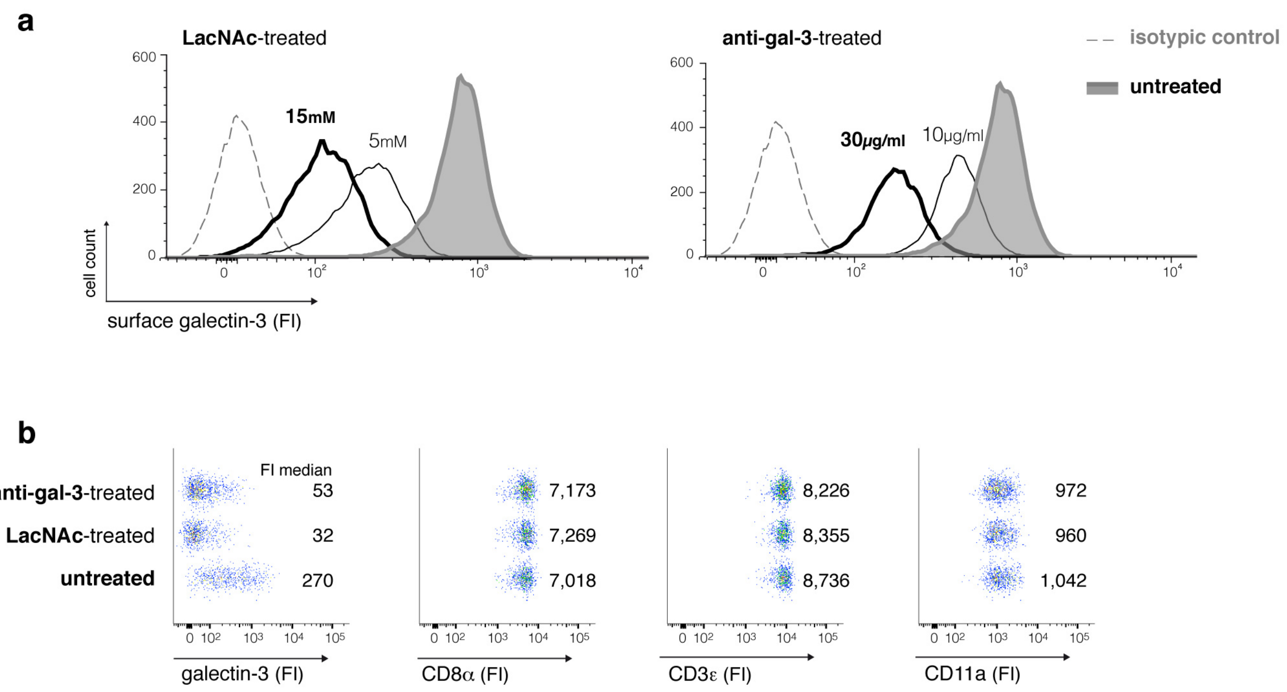

Supplementary Figure 1. **Treatment with LacNAc or an anti-galectin-3 antibody detaches galectin-3 from TIL surface**

(**a**) Total cells of ascite from patient Gdc55 *(Supplementary Table 1)* were treated for 2 h with LacNAc (*left panel*) or an anti-galectin-3 antibody (*right panel*, Mabtech) at the indicated doses. Cells were washed before being stained with anti-galectin-3 (Clone M3/38) and anti-CD8β antibodies. Graphs represent galectin-3 staining at the surface of $CD8^+$ TILs. (**b**) Total cells of ascites from patient LB3343 *(Supplementary Table 1)* were treated for 2 h with LacNAc (5 mM) or anti-galectin-3 antibody (10 µg/ml, Mabtech). Graphs represent galectin-3 staining, CD8α, CD3ε and CD11a expression at the surface of $CD8^+$ $CD3^+$ TILs. The fluorescence intensity (FI) median is indicated. Results are from one representative sample out of three.

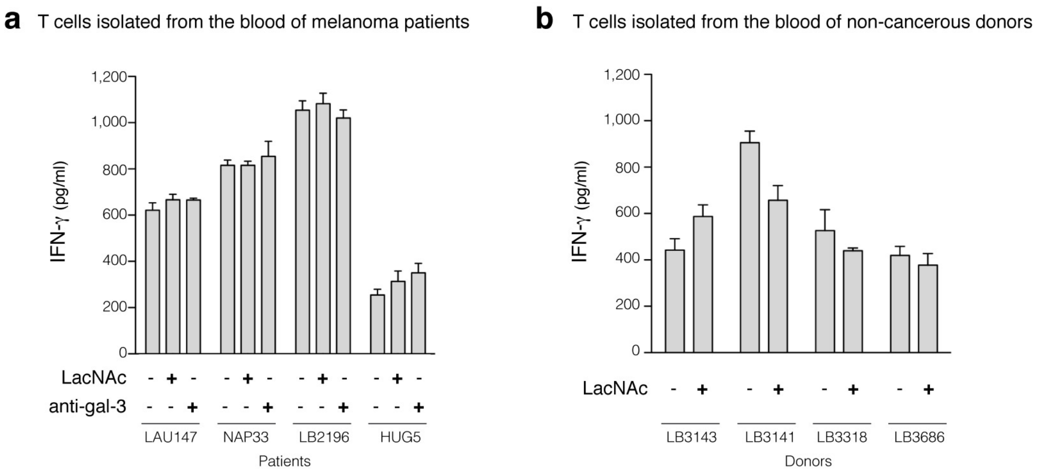

Supplementary Figure 2. **LacNAc treatment does not improve IFN-γ secretion by blood T cells**

CD8 T cells were isolated from the blood of (**a**), melanoma-bearing patients or (**b**), non-cancerous donors and treated with LacNAc or anti-galectin-3 mAb. Ten thousand T cells were cultured for 20 h with sAg-pulsed EBV-B cells. IFN-γ secretion was measured by ELISA. Columns represent mean ± SD of triplicates. Results are from one (**a**) and three (**b**) independent experiments.

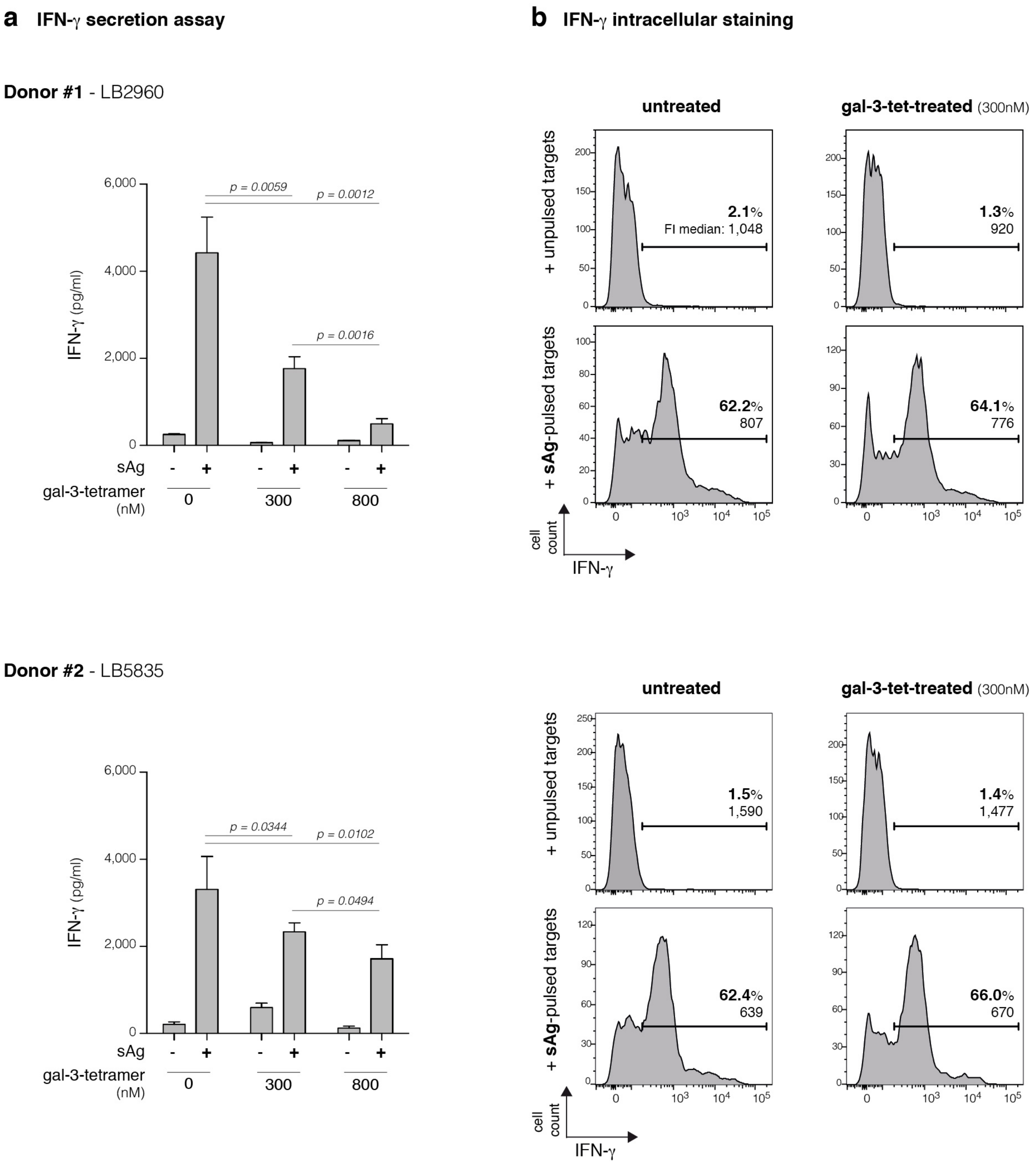

Supplementary Figure 3. **Effect of galectin-3 loading on blood CD8 T cells**

Blood CD8 T cells from non-cancerous donors LB2960 and LB5835 were cocultured with anti-CD3/CD28 coated beads at ratio 3:1, in presence of a galectin-3 tetramer. After 3 days, T cells were washed and assessed for their ability to secrete or produce IFN-γ upon stimulation with sAg-pulsed targets. (**a**) Ten thousand T cells were cultured with sAg-pulsed EBV-B cells at ratio 1:1. IFN-γ secretion was measured by ELISA. Columns represent means ± SD of triplicates; unpaired *t* test. (**b**) Fifty thousand T cells were cultured with sAg-pulsed EBV-B cells at ratio 1:1 in presence of brefeldin A. After 20 h, cells were stained for intracellular IFN-γ. The % of IFN-γ^+ TILs and the fluorescence intensity (FI) median of the positive subset are indicated. Similar IFN-γ secretion results were obtained in another experiment.

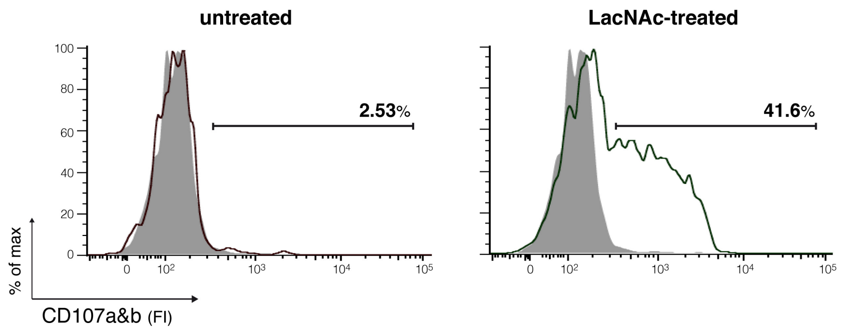

***Supplementary Figure 4*. LacNAc treatment improves the ability of TILs to degranulate**

CD8 T cells were isolated from patient Gdc55 *(Supplementary Table 1)*, treated with LacNAc and cultured with sAg-pulsed EBV-B cells in presence of FITC-coupled anti-CD107a and CD107b mAbs (*black*) or control isotypes (*grey*) and brefeldin A. After 5 h of coculture, cells were stained with an anti-CD8β. Graphs show the percentage of CD107 positive events for the CD8β^+ TIL subsets. Results are from one representative experiment out of three.

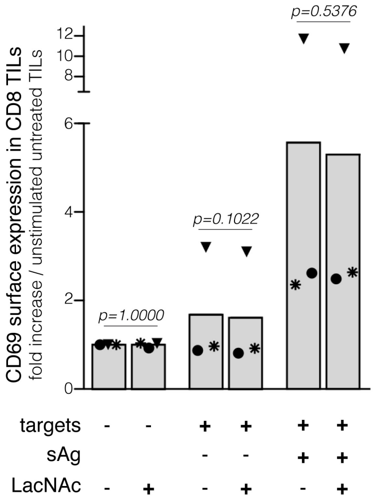

***Supplementary Figure 5*. LacNAc treatment is dispensable for CD69 upregulation**

CD8 TILs were isolated from ascites, treated with LacNAc and cultured with sAg-pulsed EBV-B cells for 5 h. Cells were stained with an anti-CD69 mAb. Data represent CD69 surface expression on TILs, relative to unstimulated conditions. Each symbol represents TILs from one patient *(Supplementary Table 1)*. Columns represent means for several patients. Paired *t* test. Results are from two independent experiments.

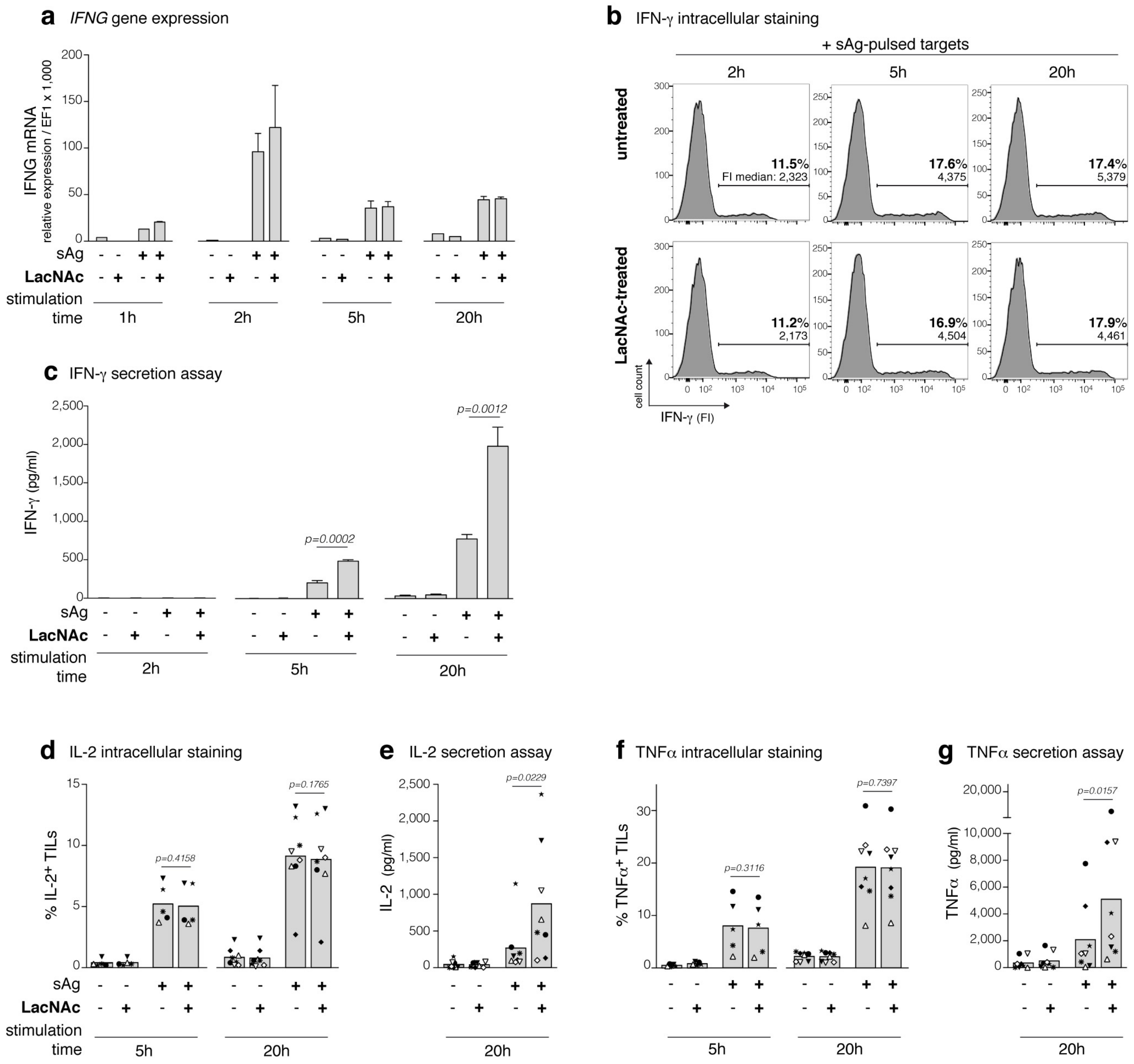

***Supplementary Figure 6*. LacNAc treatment is dispensable for cytokine production by TILs**

(**a**-**c**) CD8 TILs from sample Gdc79 (Supplementary Table 1) were treated with LacNAc and cultured with sAg-pulsed EBV-B cells. Results are from one representative experiment out of two. (**a**) IFN-γ mRNA expression was analyzed by quantitative RTqPCR. Columns represent the mean ± SD of duplicates. (**b**) Intracellular staining of IFN-γ in TILs co-cultured in the presence of brefeldin A. % of IFN-γ^+ TILs and the fluorescence intensity (FI) median of the positive subset are indicated. (**c**) IFN-γ secretion was measured by ELISA. Columns represent the mean ± SD of triplicates. (**d**-**g**) CD8 TILs were isolated from ascites and treated with LacNAc. TILs were cultured with sAg-pulsed EBV-B cells at the indicated time points. Each symbol represents TILs from one patient *(Supplementary Table 1)*. Graphs represent the % of TILs positive for (**d**), IL-2 and (**e**), TNF-α. (**f**), IL-2 and (**g**), TNF-α secretions were measured by Bioplex.

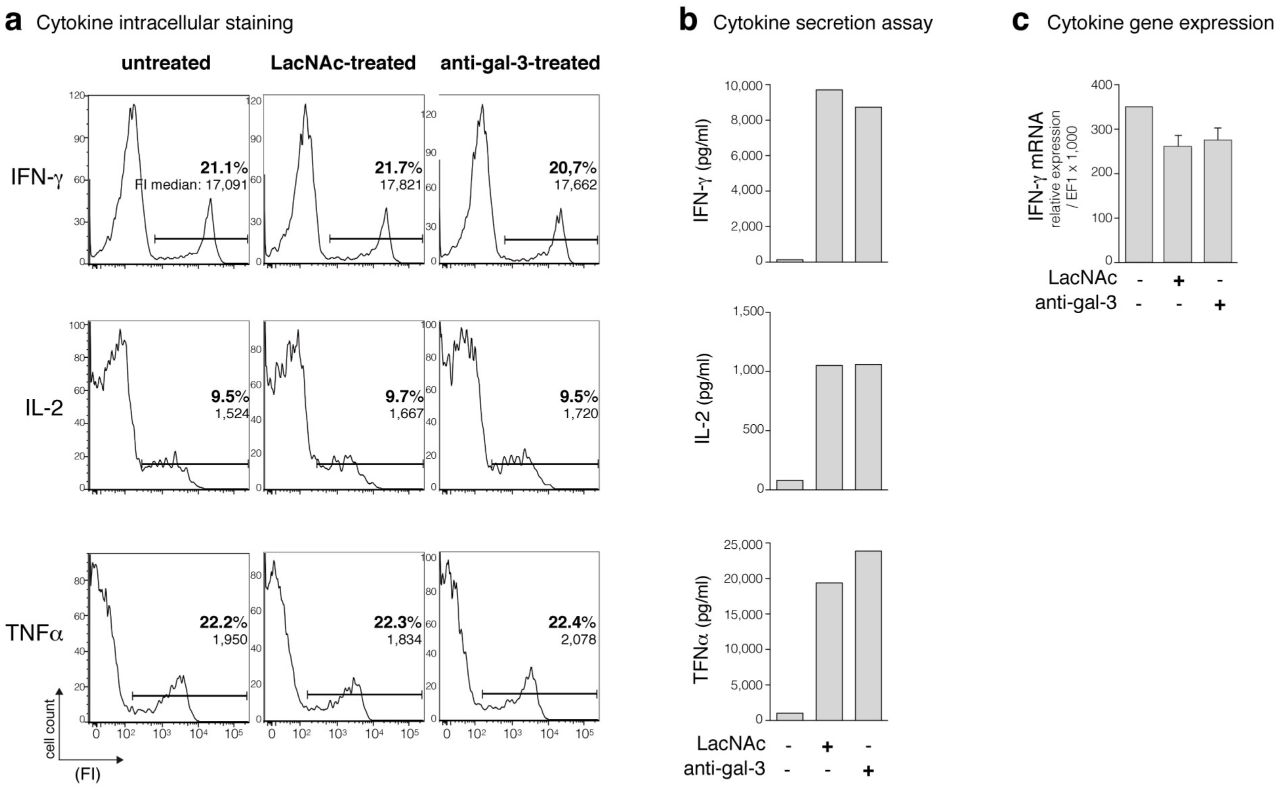

Supplementary Figure 7. **Treatment with an anti-galectin-3 antibody is dispensable for cytokine production by TILs**

CD8 TILs from sample Gdc31b (Supplementary Table 1) were treated with LacNAc or an anti-galectin-3 mAb and co-cultured with sAg-pulsed EBV-B cells. (**a**) Intracellular staining of IFN-γ, IL-2 and TNF-α in TILs stimulated for 20 h in the presence of brefeldin A. Representative histograms, % of IFN-γ^+ TILs and the fluorescence intensity (FI) median of the positive subset are indicated. (**b**) After 20 h of coculture, cytokine secretion was measured by Bioplex. (**c**) IFN-γ mRNA expression was measured by quantitative RT-PCR on sample collected after 2h of co-culture.

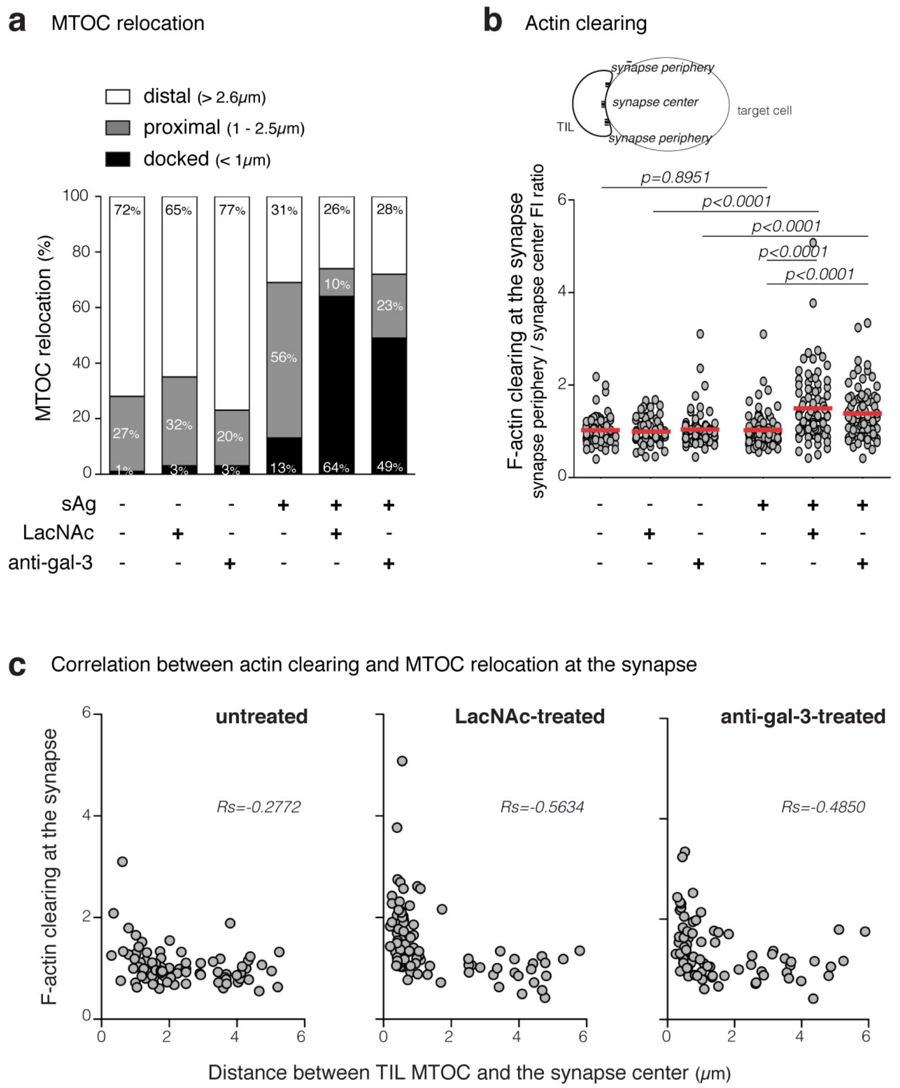

***Supplementary Figure 8*. Treatment of TILs with an anti-galectin-3 antibody favors secretory synapse completion**

CD8 TILs were obtained from sample Gdc31b *(Supplementary Table 1)*. TILs were treated with LacNAc or an anti-galectin-3 antibody and conjugated with sAg-pulsed EBV-B cells, previously loaded with CMTMR. After 25 min, cells were fixed, permeabilized and stained for α-tubulin and F-actin. (**a**) MTOC polarization was evaluated by measuring the distance between MTOC and the center of the TIL-target contact zone; The MTOC was considered as "docked" to the membrane when it was closer than 1 μm of the center of the contact zone, as "proximal" when it was located between 1 and 2.5 μm, and as "distal" when at more than 2.6 μm. n>85. (**b**) Actin clearing at the synapse center was evaluated by comparing fluorescence intensities (FI) at the synapse periphery and the synapse center. Each dot corresponds to a TIL-target conjugate; n>85; Mann-Whitney U test. (**c**) Correlation between actin clearing and MTOC polarization at the synapse center; n>85. Spearman's rank correlation coefficients (Rs) are indicated.

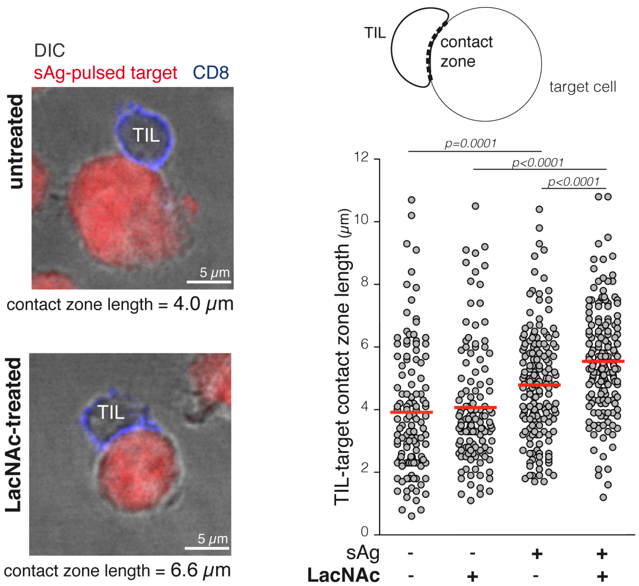

***Supplementary Figure 9*. LacNAc treatment improves TIL spreading on their targets**

CD8 TILs from patient Gdc31a *(Supplementary Table 1)* were treated with LacNAc and conjugated with sAg-pulsed EBV-B cells, previously loaded with CMTMR (*red*). After 25 min, cells were stained for CD8α (*blue*) and observed by confocal microscopy. The length of the TIL-target contact zone was measured as depicted. Each dot represents one conjugate; n>123; unpaired *t* test. Results are from one representative experiment out of two. *DIC, differential interference contrast.*

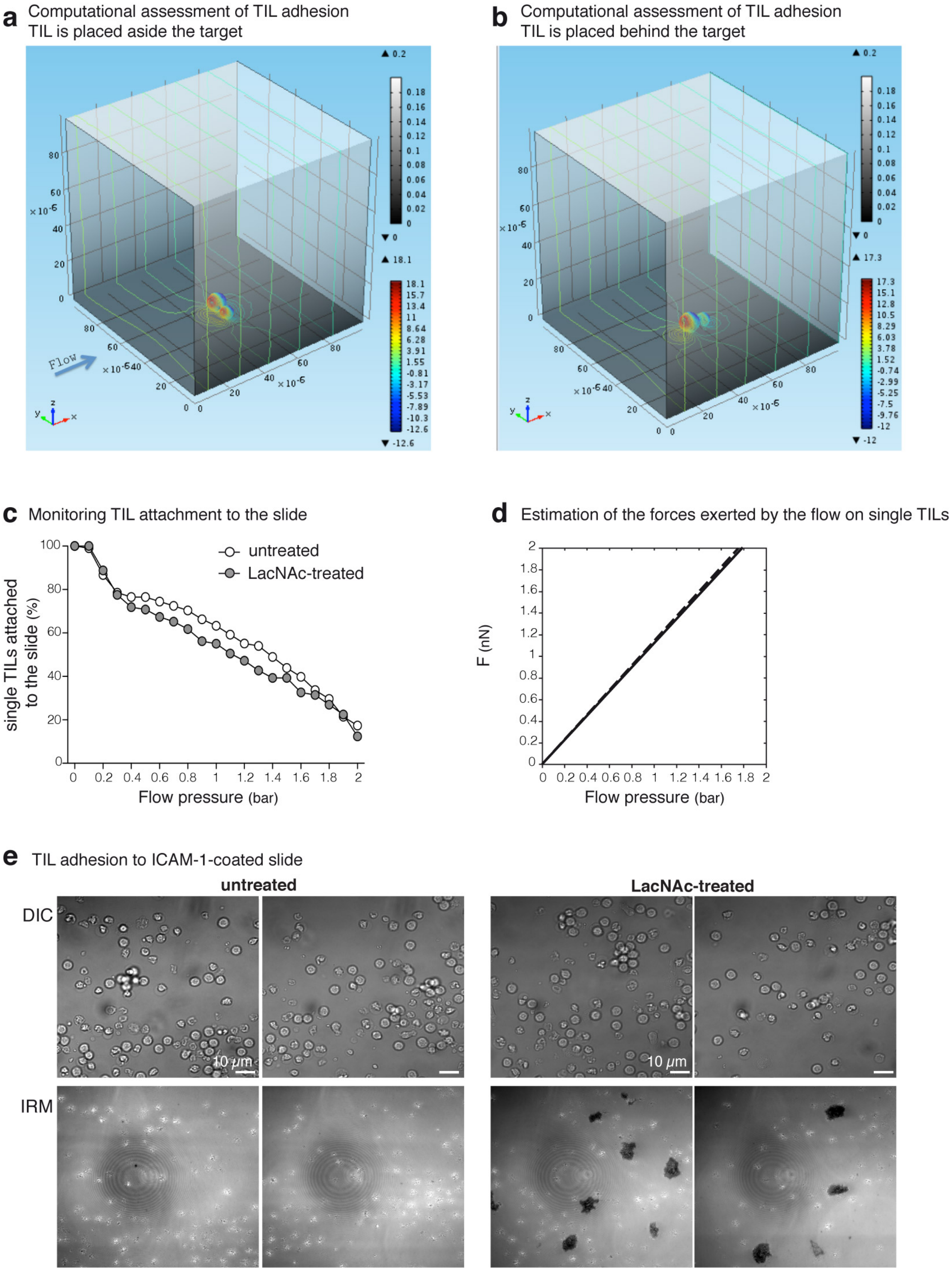

Supplementary Figure 10. **TIL adhesion**

CD8 TILs were obtained from patients (**a**-**d**), LB3450 and (**e**), Gdc58 *(Supplementary Table 1)*. (**a**,**b**) Computational domain with dimensions in meter (m). The grey levels represent the magnitude of the flow velocity component in the x-direction (m s^{-1}) and the colored contours represent the pressure field (Pa), taken relative to the outlet pressure. (**a**), TIL is placed aside the target (**b**), TIL is placed behind the target. (**c**) Detachment of single TILs was examined. n>88. (**d**) Forces exerted by the flow on single TILs of 6.5 μm of diameter (*solid line*) together with the O'Neill's analytical solution (*dashed line*). (**e**) TILs were treated with LacNAc and were allowed to settle on ICAM-1-coated slides. After 25 min, cells were observed by interference reflection microscopy (IRM). Representative images are from one experiment out of two.

a Fluorescence loss in photobleaching measurements

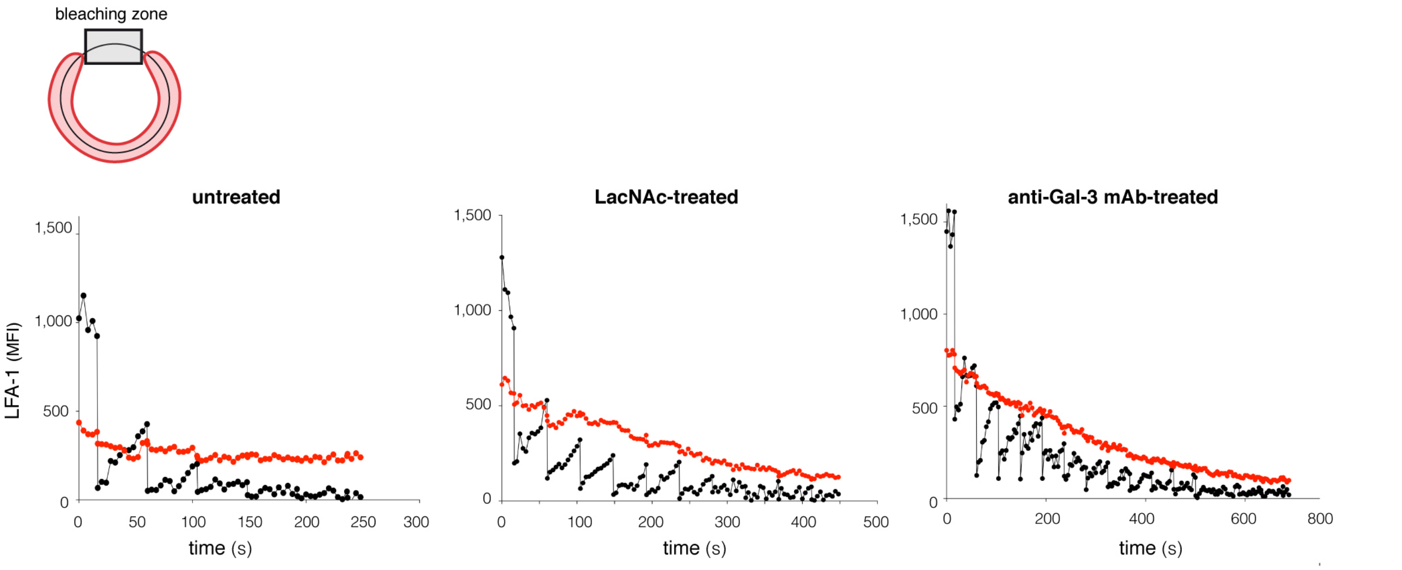

b Estimation of LFA-1 mobile fraction

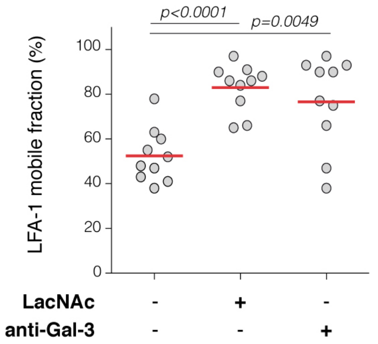

Supplementary Figure 11. **LFA-1 mobility at the surface of CD8 TILs**

CD8 TILs were isolated from ascites obtained from patient LB3390 (Supplementary Table 1) and treated with LacNAc or anti-galectin-3 antibody (Mabtech). Cells were stained with an AlexaFluor 488-labeled anti-CD11a antibody (HI111; Biolegend). LFA-1 mobile fraction was estimated by using *Fluorescence Loss in Photobleaching* (FLIP) analysis. (**a**) The extent of fluorescence loss was monitored at distant regions (*red*) from the beaching zone (*black*). (**b**) Fluorescence intensities in the measurement region were plotted against time and normalized between 0 and 100%, zero was defined as the smallest value in the data set, 100 as the largest value in the data set. The immobile fraction is determined by the mean of the five lowest intensities. The mobile fraction = 1/immobile fraction. Each dot corresponds to a TIL; unpaired *t* test.

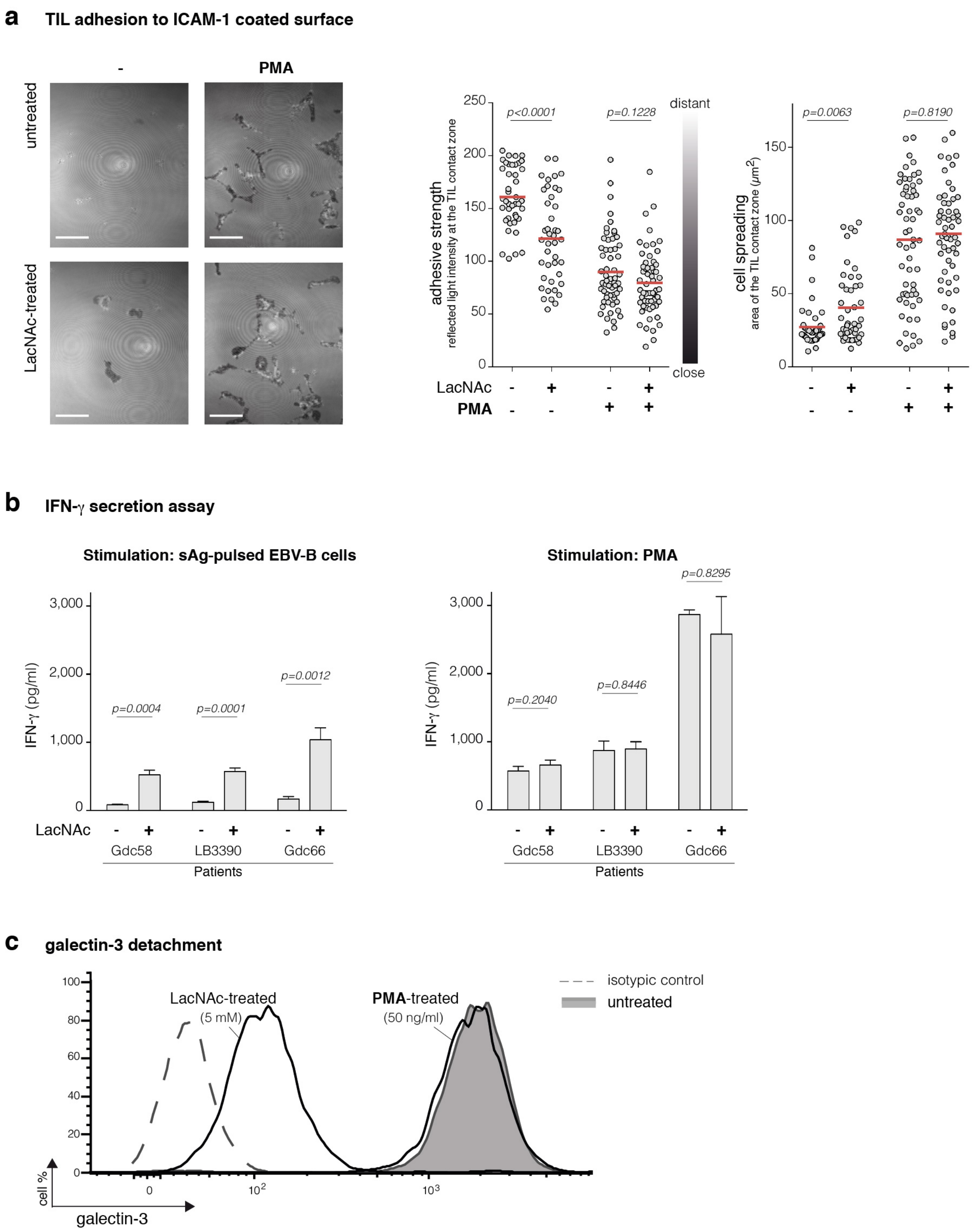

***Supplementary Figure 12*. PMA stimulation rescues the adhesive and secretory defects of TILs covered with galectins**

CD8 TILs were isolated from sample Gdc58 (**a**,**b**), LB3390 and Gdc66 (**b**), and were treated with LacNAc. (**a**) TILs were allowed to settle on ICAM-1-coated slides in presence of PMA (50 ng/ml). After 25 min, cells were observed by IRM. Scale bars represents 10 μm. Quantification of the intensity of the reflected light and of the area of the T cell contact zone. n>45; Mann-Whitney U test. (**b**) CD8 TILs were cultured with sAg-pulsed EBV-B cells or in presence of PMA. After 20 h, IFN-γ secretion was measured by ELISA. Columns represent means ± SD of triplicates; unpaired *t* test. Results are from one representative experiments out of two. (c) Total cells of ascite from patient Gdc55 (Supplementary Table 1) were treated for 2 h with PMA or LacNAc at the indicated doses. Cells were washed before being stained with anti-galectin-3 (Clone M3/38) and anti-CD8ß antibodies. Graphs represent galectin-3 staining at the surface of CD8+ TILs.

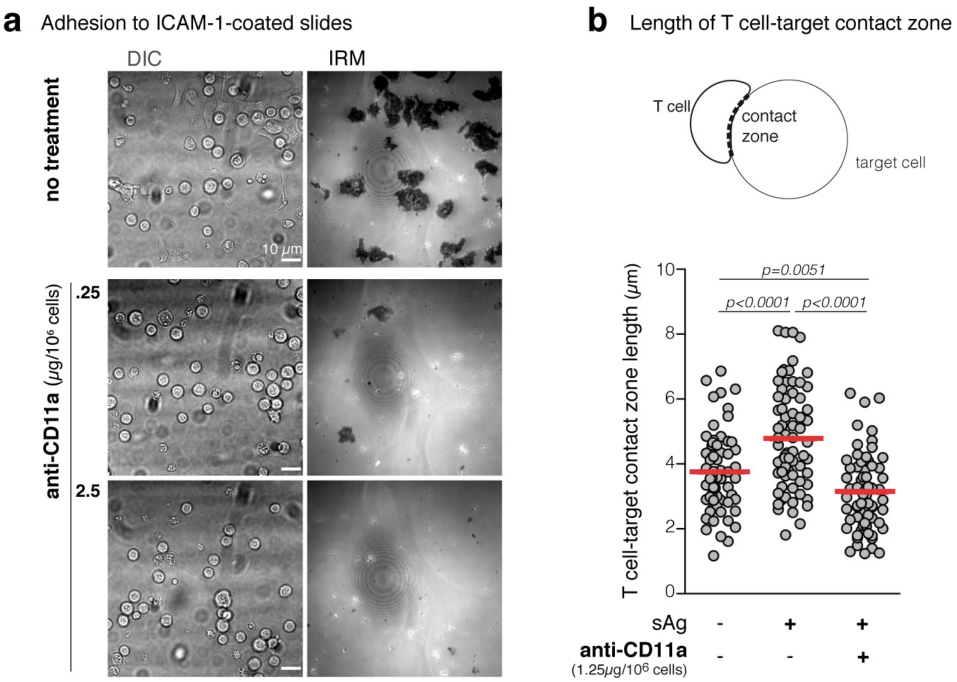

***Supplementary Figure 13*. Blocking LFA-1 in functional CD8 blood T cells**

Blood CD8 T cells from non-cancerous donor LB2050 were treated with an anti-CD11a blocking antibody at the indicated doses. (**a**) T cells were allowed to settle on ICAM-1-coated slides. After 25 min, T cells were observed by *Interference Reflection Microscopy* (IRM). (**b**) T cells were conjugated for 25 min with sAg-pulsed EBV-B cells. The length of the TIL-target contact zone was measured as depicted. Each dot corresponds to a T cell-target conjugate, n>60; unpaired *t* test. *DIC, differential interference contrast.*

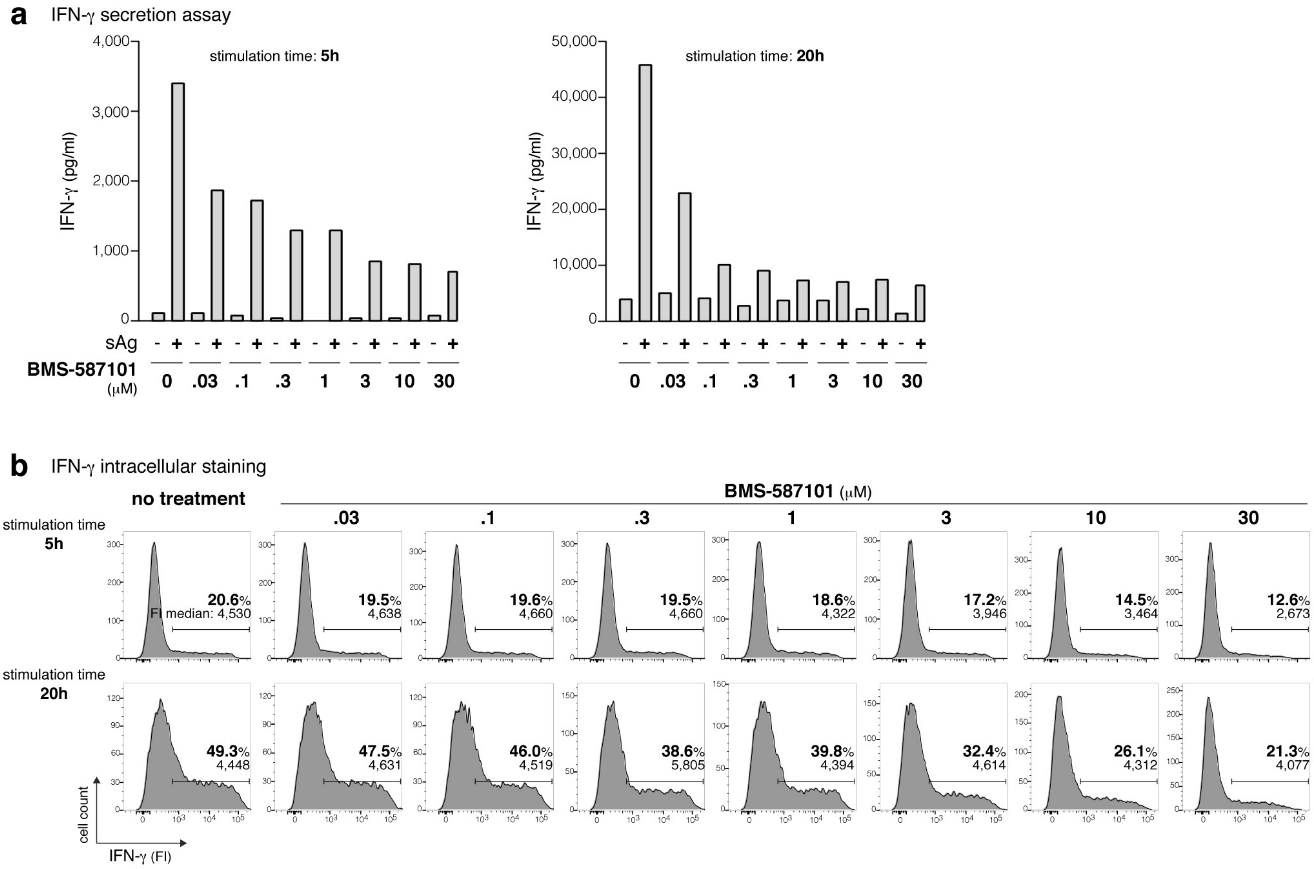

***Supplementary Figure 14*. Effect of the LFA-1 small molecule antagonist BMS-587101 on the secretion and the intracellular production of IFN-γ**

Blood CD8 T cells from non-cancerous donor LB5739 were treated with LFA-1 small molecule antagonist BMS-587101 and stimulated with sAg-pulsed B cells. (**a**) After 5-20 h of stimulation, IFN-γ secretion was analyzed by ELISA. (**b**) After 5-20 h of stimulation in the presence of brefeldin A, cells were stained for intracellular IFN-γ. % of IFN-γ^+ T cells and FI medians of the positive subsets are indicated. Results are from one representative experiment out of three.

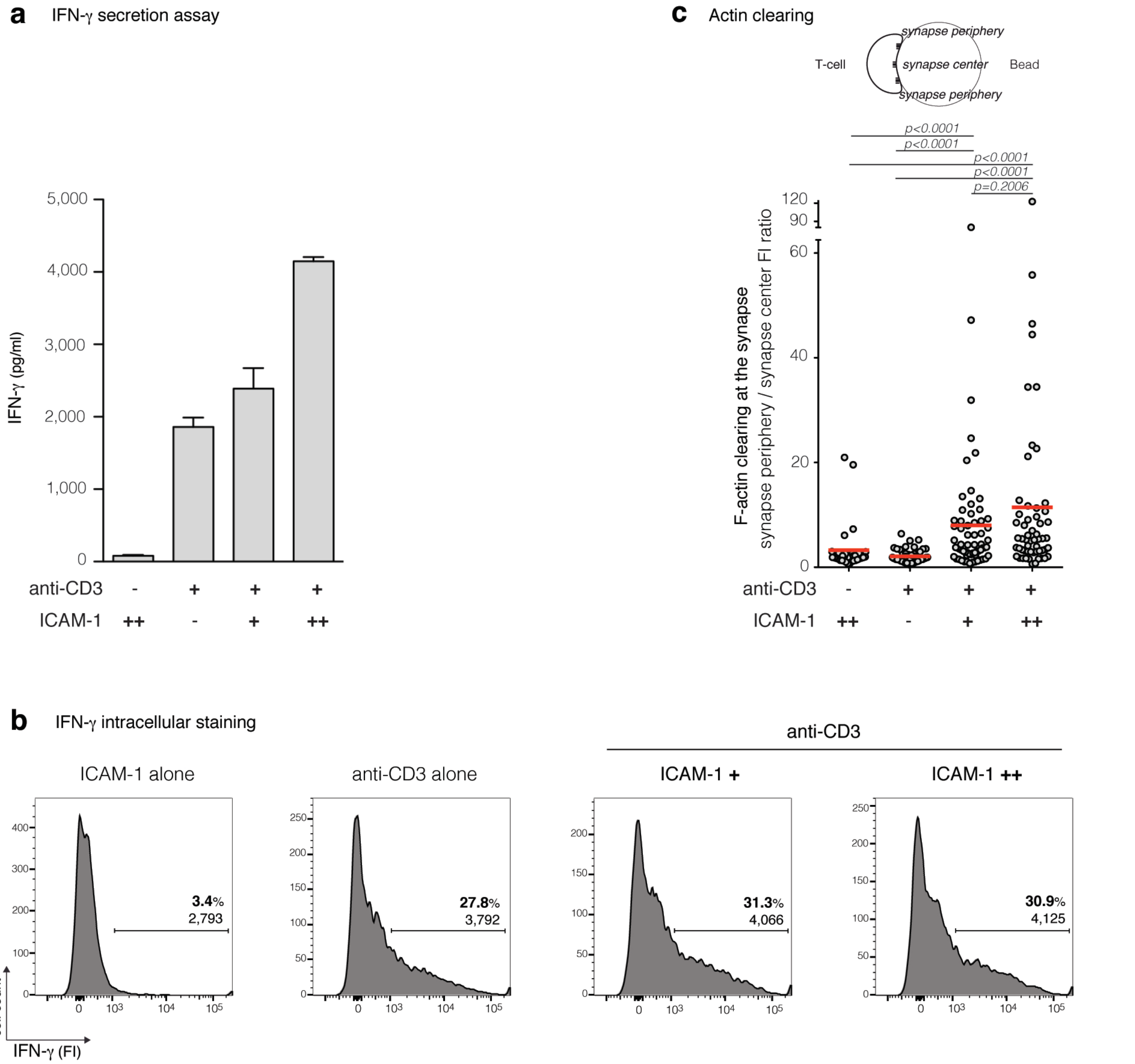

***Supplementary Figure 15*. Function of blood CD8 T cells stimulated by anti-CD3 and ICAM-1-coated beads**

CD8 blood T cells from non-cancerous donor LB3433 were co-cultured with Protein A Magnetic beads (Merck Millipore, Temecula, CA, USA) previously coated with anti-CD3 (clone OKT3, Mabtech, 4.2 pg/bead) and recombinant human ICAM-1-Fc (R&D Systems, 1.6 pg/bead (**+**) or 14.2 pg/bead (**++**) and subsequently blocked with mice IgG2b antibodies. (**a**) IFN-γ secretion was measured by ELISA after overnight co-culture. Columns represent the mean ± SD of duplicates. (**b**) Intracellular staining of IFN-γ in T cells co-cultured overnight in the presence of brefeldin. Percentages of IFN-γ^+ TILs and the fluorescence intensity (FI) median of the positive subset are indicated. **(c)** After 25 min, cells were stained for actin (*green*) with AlexaFluor 488 phalloidin (Molecular Probes). Cells were analyzed using a Zeiss LSM 510 confocal microscope with a 63x NA1.4 Plan-Apochromat oil immersion objective. The intensity of F-actin staining was measured using the Linescan Function of software MetaMorph. Actin clearing at the synapse center was evaluated by comparing fluorescence intensities (FI) at the synapse periphery and the synapse center. Each dot corresponds to a T cell-bead conjugate; n > 30; Mann-Whitney U test. Results are from one representative experiment out of two.

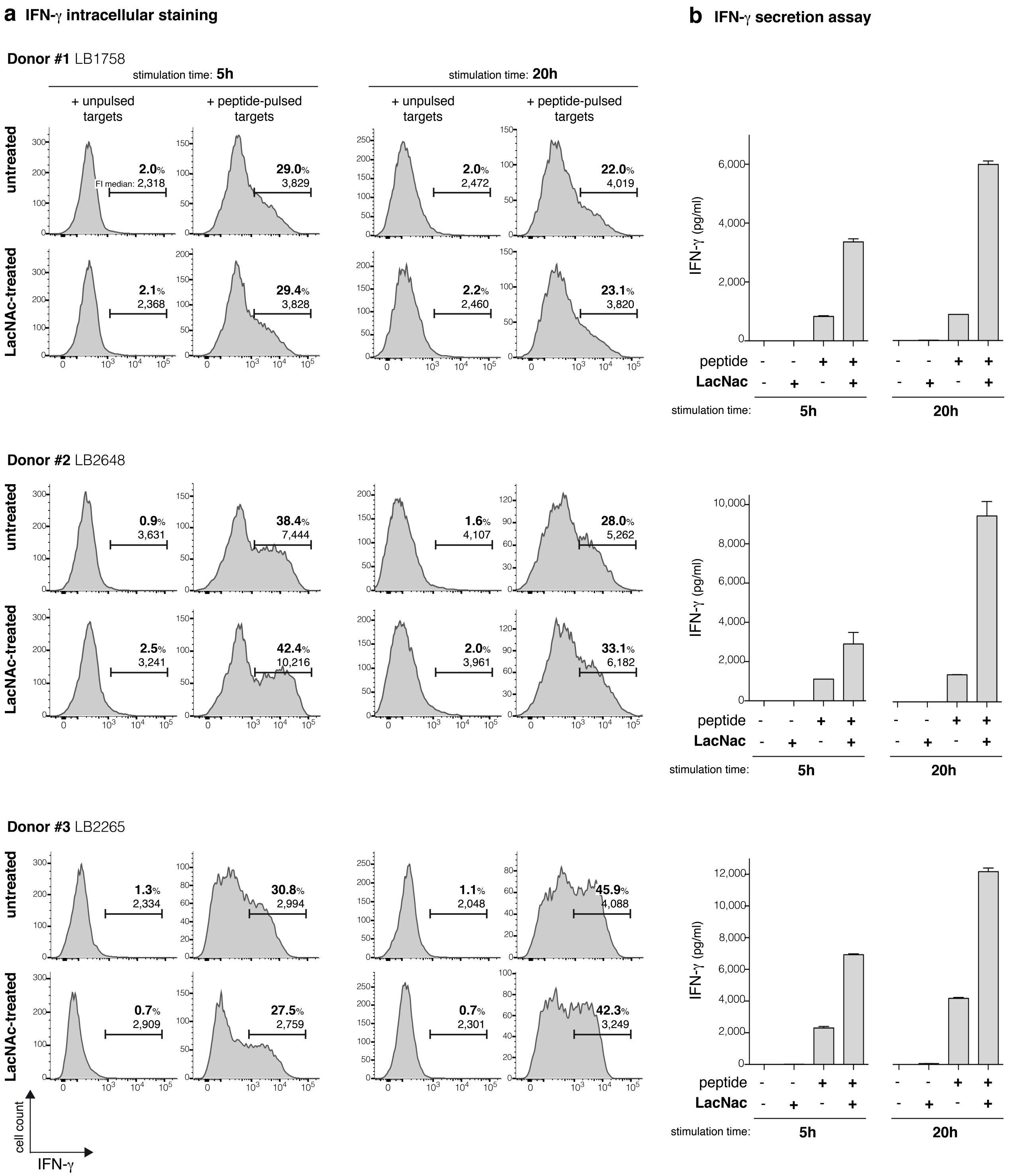

Supplementary Figure 16. **LacNAc treatment improves IFN-γ secretion by polyclonal CD8 T cells specific for a Melan-A peptide presented by HLA-A2**

CD8 T cells were sorted from blood samples from three donors with a fluorescent HLA-A2/Melan-A multimer and expanded *in vitro* by regular stimulations with autologous mature HLA-A2 dendritic cells pulsed with Melan-A peptide EAAGIGILTV at 1 μM (two stimulations), and subsequently with cells from an HLA-A2 melanoma cell line expressing Melan-A (CP50-MEL) and LG2-EBV feeder cells (two stimulations). These CD8 T cells were incubated with T2 targets pulsed or not with 1 μM of peptide, at 1:1 ratio. (**a**) Intracellular staining of IFN-γ in TILs co-cultured in the presence of brefeldin. Percentages of IFN-γ^+ TILs and the fluorescence intensity (FI) median of the positive subset are indicated. (**b**) IFN-γ secretion was measured by ELISA. Columns represent the mean ± SD of duplicates.

Cancer type	Patient code	IFN-γ secretion (pg/ml)				
		Untreated TILs		LacNAc-treated TILs		Fold change
		Mean of triplicates	± SD	Mean of triplicates	± SD	
Pancreatic adenocarcinoma	Gdc13 ★	526	*±62*	2,277	*±150*	**4.3**
	Gdc29 ▲	427	*±13*	1,707	*±373*	**4.0**
	Gdc31a ▼	691	*±106*	1,785	*±206*	**2.5**
	Gdc31b ▽	404	*±24*	10,001	*±0*	**24.8**
	Gdc44 ●	991	*±170*	3,015	*±601*	**3.0**
	Gdc55 ◇	192	*±65*	809	*±188*	**4.2**
	Gdc79	2,381	*±55*	4,490	*±879*	1.9
	Gdc80 ◖	464	*±35*	2,725	*±1,218*	**5.8**
	LB3377	117	*±29*	509	*±47*	**4.3**
	LB3416	479	*±106*	8,516	*±1300*	**17.8**
	LB3426	1,105	*±31*	6,506	*±365*	**5.9**
Colon adenocarcinoma	Gdc35	65	*±13*	356	*±37*	**5.5**
	Gdc42 △	157	*±7*	2,095	*±47*	**13.3**
	Gdc54	3,712	*±892*	4,001	*±649*	1.1
	Gdc63	573	*±92*	1,714	*±93*	**3.0**
	Gdc64	70	*±4*	452	*±45*	**6.5**
	LB3395	1,301	*±205*	2,402	*±30*	1.8
Cholangiocarcinoma	Gdc26	340	*±31*	1,627	*±368*	**4.8**
	Gdc45	20	*±14*	50	*±10*	**2.5**
	LB3450 □	121	*±13*	384	*±45*	**3.2**
Gastric adenocarcinoma	LB3200	1,335	*±301*	3,721	*±752*	**2.8**
	Gdc48a ✳	166	*±16*	698	*±100*	**4.2**
	Gdc48b	245	*±71*	1,182	*±47*	**4.8**
Oesophageal adenocarcinoma	Gdc33	114	*±7*	557	*±212*	**4.8**
Ovarian adenocarcinoma	LB2992	513	*±44*	947	*±233*	1.8
	LB3045 ■	603	*±69*	1,774	*±101*	**2.9**
	LB3131	114	*±7*	529	*±48*	**4.6**
	LB3390	16	*±21*	329	*±34*	**20.6**
	LB3427	633	*±147*	2,259	*±19*	**3.6**
	VUB190	221	*±17*	568	*±40*	**2.6**
Endometrial adenocarcinoma	Gdc61	83	*±20*	208	*±20*	**2.5**
Chronic lymphocytic leukemia	Gdc58 ◆	605	*±66*	1,493	*±109*	**2.5**
Bronchial adenocarcinoma	LB3411 ○	84	*±5*	553	*±21*	**6.6**
Head and neck squamous carcinoma	Gdc57	66	*±2*	425	*±48*	**6.4**
Invasive ductal carcinoma	Gdc37	98	*±7*	939	*±176*	**9.5**
	Gdc66	177	*±36*	1,060	*±74*	**6.0**
	LB5670	120	*±41*	658	*±233*	**5.5**
Breast lobular carcinoma	Gdc68	140	*±16*	1,409	*±620*	**10.0**
Melanoma	LB5669	420	*±82*	1,081	*±71*	**2.6**
Neoplasia unknown origin	Gdc38	92	*±6*	214	*±27*	**2.3**

Supplementary Table 1. **LacNAc treatment boosts IFN-γ secretion by CD8 TILs isolated from ascites associated with different malignancies**

CD8 TILs were isolated from malignant ascites or pleural effusions, and treated overnight with LacNAc. Ten thousand T cells were cultured for 20 h with sAg-pulsed EBV-B cells. IFN-γ secretion was measured by ELISA. Values are mean ± SD of triplicates. Ascites collected at different time points from the same patient are indicated by a/b. Selected TIL sample is represented by a unique symbol in the figures.
